# Host–guest complexes between cryptophane-C and chloromethanes revisited

**DOI:** 10.1002/mrc.3898

**Published:** 2012-11-07

**Authors:** Z Takacs, M Soltesova, J Kowalewski, J Lang, T Brotin, J-P Dutasta

**Affiliations:** a Arrhenius Laboratory, Department of Materials and Environmental Chemistry, Stockholm UniversitySE-106 91, Stockholm, Sweden; b Faculty of Mathematics and Physics, Department of Low Temperature Physics, Charles University in PragueV Holesovickach 2, CZ-180 00, Prague 8, Czech Republic; c Laboratoire de Chimie, (CNRS-UMR 5182) Ecole Normale Superieure de Lyon46 Allée d'Italie, F-69364, Lyon cedex 07, France

**Keywords:** inclusion complexes, complexation kinetics, cross relaxation

## Abstract

Cryptophane-C is composed of two nonequivalent cyclotribenzylene caps, one of which contains methoxy group substituents on the phenyl rings. The two caps are connected by three OCH_2_CH_2_O linkers in an *anti* arrangement. Host–guest complexes of cryptophane-C with dichloromethane and chloroform in solution were investigated in detail by nuclear magnetic resonance techniques and density functional theory (DFT) calculations. Variable temperature proton and carbon-13 spectra show a variety of dynamic processes, such as guest exchange and host conformational transitions. The guest exchange was studied quantitatively by exchange spectroscopy measurements or by line-shape analysis. The conformational preferences of the guest-containing host were interpreted through cross-relaxation measurements, providing evidence of the gauche+2 and gauche−2 conformations of the linkers. In addition, the mobility of the chloroform guest inside the cavity was studied by carbon-13 relaxation experiments. Combining different types of evidence led to a detailed picture of molecular recognition, interpreted in terms of conformational selection. Copyright © 2012 John Wiley & Sons, Ltd.

## Introduction

Cryptophanes are molecular hosts, able to bind small organic guests (such as chloroform or dichloromethane) inside their cavity. Cryptophanes were first prepared and characterized by Collet *et al*.^1^–^10^ in the 1980s. We have reported over the last decade a series of nuclear magnetic resonance (NMR) studies of cryptophane complexes with chloroform and dichloromethane.^11^–^18^ Among other physicochemical studies of cryptophane complexes of relevance for the present work, we wish to mention the vibrational dichroism studies combined with quantum chemical calculations of the DFT type.^19^–^21^ The chemistry of cryptophanes has been subject to several reviews, the most recent published in 2009.^22^

**Scheme 1:**
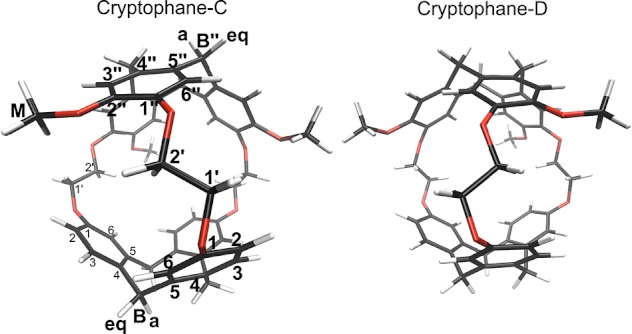
Structures of the *T*_1_*T*_1_*T*_1_ conformers of cryptophane-C and D.

Cryptophanes consist of two cyclotribenzylene (CTB) units (denoted as the caps), connected by three linkers, commonly of the –O-(CH_2_)*_n_*-O– type. A group of early synthesized compounds, called cryptophane-A, cryptophane-C and cryptophane-D, corresponds to *n* = 2. Cryptophane-A has two identical caps, carrying a methoxy group on each phenyl group. Cryptophanes C and D have the caps different—one carrying the methoxy substituents on the phenyl groups and one containing unsubstituted phenyl units (see Scheme 1). They are diastereoisomers—cryptophane-C is the *anti* isomer (in analogy with cryptophane-A) and cryptophane-D is the *syn* isomer. Both of these systems were recently studied by our laboratories. For cryptophane-C, we reported a ^1^H NMR study of the complex with chloroform, including measurements of cross relaxation between bound chloroform and host protons, where the most important finding was that one of the orientations of the guest molecule inside the host cavity was somewhat more probable than the other one.^17^ Cryptophane-D was studied in complexes with both chloroform and dichloromethane, using both the ^1^H and ^13^C NMR spectroscopy, along with quantum chemical (DFT) calculations.^18^ Some of the finding of that work—not least the improved understanding of the conformational equilibria for the free and bound host—motivated us to return to the cryptophane-C as host. In this communication, we include both chloroform and dichloromethane as guests, use both ^1^H and ^13^C NMR spectroscopic tools and correct some errors in the previous study.^17^

The outline of this article is as follows. The experimental details are provided in the next section. The results, spectroscopic as well as computational, are presented in the section to follow. The conclusions are drawn in the final section.

## Experimental

### Materials and sample preparations

Cryptophane-C was synthesized according to a procedure described by Brotin *et al*.2005 The reaction gives a mixture of cryptophane-D and cryptophane-C, which are separated by column chromatography. The material was recrystallized in chloroform–ethanol mixture and then washed several times with diethyl ether. The raw material thus contained a small amount of these solvents. The solid-solution extraction method was used to obtain cryptophane-C free of all solvents and potential guests. The raw cryptophane-C powder was dipped into tetrachloromethane at least once, and volatile species were let to evaporate. The advantage of this method is that cryptophane-C is not soluble in tetrachloromethane but the solvents are. The tetrachloromethane molecule (molecular volume of 86.7 Å^3^ estimated following Zhao *et al*.2003) is too big to enter the cryptophane-C cavity, and the remaining CCl_4_ in the powder does not compete with other guests. 1,1,2,2-Tetrachloroethane-d_2_ was used as a solvent for all samples. We observed that cryptophane-C displays a lower solubility in tetrachloroethane (max. 13 mM) than cryptophane-D.

The following samples were prepared:

1. 11 mM cryptophane-C: this sample was not used for quantitative experiments and was not subject to extraction with CCl_4_.
2. 11 mM cryptophane-C and 13.8 mM CH_2_Cl_2_.
3. 11 mM cryptophane-C and 88 mM CH_2_Cl_2_ (an earlier version of purification procedure using CH_2_Cl_2_ instead of CCl_4_ was used for this sample 18).
4. 10 mM cryptophane-C and 50 mM labeled ^13^CHCl_3_ for the quantitative NMR relaxation and chemical exchange measurements.
5. three samples of 7 mM cryptophane-C with 9 mM, 13 mM and 25 mM nonlabeled CHCl_3_, respectively, for accurate determination of the association constant.
6. 10 mM of cryptophane-C and 10 and 50 mM CHCl_3_, respectively, without the purification procedure, for qualitative measurements only.

^13^C labeled chloroform and the deuterated solvent, 1,1,2,2-tetrachloroethane-d_2_, were obtained from Cambridge Isotope Laboratories. Nonlabeled chloroform was obtained from VWR and dichloromethane from Scharlau Chemie S.A.

### NMR spectra

^1^H and ^13^C spectra were recorded with Bruker Avance spectrometers operating at 9.4, 14.1 and 16.5 T using 5 mm (BBI and BBO at 9.4 T, TXI and BBO at 14.1 T and cryo-TXI at 16.5 T) probe heads. At 9.4 T, the temperature calibration was performed using standard methanol calibration sample, and a resistance detector made of copper wire dipped into silicon oil contained in a 5-mm NMR tube was used at the two higher fields. The accuracy of the temperature determination is estimated at ±1 K. All the NMR experiments used for quantitative evaluation of build-ups and decays were repeated at least twice. The peak assignment was based on DQF-COSY, [two-dimensional (2D)] nuclear Overhauser effect spectroscopy (NOESY), (2D) rotating frame nuclear Overhauser effect spectroscopy (ROESY) and ^1^H-^13^C heteronuclear single quantum correlation (HSQC) and heteronuclear multiple bond correlation (HMBC) experiments.

On the TXI probe at 14.1 T, the nutation frequency for ^1^H and ^13^C were 32.7 and 22.7 kHz, respectively. The nutation frequencies on the BBO probe at 14.1 T were 18.8 and 32.5 kHz for ^1^H and ^13^C, respectively. At 16.5 T, the nutation frequencies were typically 10.3 kHz for ^1^H and 20.7 kHz for ^13^C. The BBO probe at 9.4 T used typically 24.5 kHz for ^1^H and 43.1 kHz for ^13^C, whereas the BBI probe on the same spectrometer gave 27.3 kHz for proton and 19.4 kHz for ^13^C.

The exchange spectroscopy measurements were performed in the temperature range of 280–320 K at two fields (14.1 and 16.5 T) using the implementation as the DPFGSENOE sequence with two selectively refocusing shaped pulses and one hard π pulse in the middle of the mixing time interval.^25^ The semiselective inversion pulses were implemented as Gaussian G3 cascades^26^ with the duration of 18–20 ms. Sixteen different time intervals were used. Experiments were performed with 64 accumulated signal transients, using relaxation delay of 35–40s. Only the doublets of the ^13^C-labeled chloroform were evaluated. The purpose of the label is to enhance the proton spin-lattice relaxation. The evaluation of the exchange rate of the backward (decomplexation) reaction was based on the approach proposed originally by Macura *et al*.1986 and described by Hu and Krishnamurthy.2006 The exchange rate of the forward (complexation) reaction was based on the principle of detailed balance (see following paragraphs) to avoid the error coming from small intensities at very short mixing times in the initial rate regime.^18^

^13^C spectra were recorded with Waltz16 proton decoupling at 9.4 T, and Waltz65 was used at 14.1 and 16.5 T. The decoupling power corresponded, on average, to the nutation frequency of 2.8 kHz. The spin-lattice relaxation times of the guest (^13^CHCl_3_) were measured by the inversion-recovery method using 16–19 recovery delays ranging from 0.5 ms to 30 s with a relaxation delay of 35 s. The nuclear Overhauser effect (NOE) was measured with the dynamic NOE sequence. The NOE build-up period was set to 5*T*_1_ and the relaxation delay to 10*T*_1_. In the spectrum with no NOE enhancement, the build-up period was set to 0.1 ms.

The 2D NOESY and ROESY at 9.4 T were recorded at 258 and 298 K. The detection method used was States-TPPI. The mixing times were 0.04–0.24 s for the NOESY and 0.04–0.2 s for the ROESY. The spin lock power was set to 3 kHz. In the direct dimension, eight scans were used with 16 dummy scans, with a relaxation delay of 5–7 s. The size of FID was 4096 data points, giving approximately 0.6 s acquisition time in the *t*_2_ domain. The numbers of data points acquired in the indirect (*t*^1^) dimension were 512 or 768, zero filled to 4096 points. The shifted quadratic sine bell was used as a window function in both dimensions. The NOESY pulse sequence contained a hard pulse in the middle of the mixing time accompanied by two opposite *z* gradients.^29^ The ROESY sequence used was π/2–*t*_1_–spin lock–*t*_2_.^30^,^31^ Before the data analysis, the signal-to-noise ratio was increased by adding the positive and negative projections taken at parts of the spectra where no peaks were to be found to all the rows. For data analysis, the volume integrals were used.

### Line-shape analysis

The line-shape fitting procedure was based on the Bloch–McConnell equations.^32^ Briefly, the line shape for spins (without J-coupling) undergoing exchange between a free site, *f*, and a bound site, *b*, with resonance frequencies ν*_f_* and ν*_b_*, respectively, is given by


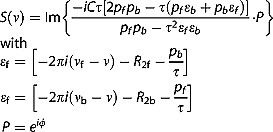
(1)

The symbols *p_f_* and *p_b_* =1 − *p_f_* denote the populations of the two sites. *C* is a scaling factor, without physical significance, *P* is a phase factor leading to absorptive line shapes, and *R*_2*f*_ and *R*_2*b*_ are the transverse relaxation rates at the two sites. The most interesting parameter is the exchange lifetime *τ*, related to the (pseudo)first-order rate constants for the free-to-bound (*k*_fb_) and bound-to-free (*k*_bf_) reactions through:


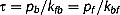
(2)

The second equality in Eqn (2) is the principle of detailed balance. The line-shape fitting was performed using an in-house script in Scilab.[Bibr b33]

### Quantum chemical calculation

All calculations were performed in a similar way as reported for cryptophane-D.^18^ The Gaussian09 package^34^ was used. Geometries were optimized at the level of DFT-B3LYP functional with the basis set 6–31 G(d). Each optimized structure was then subject to a single point energy calculation, together with the calculation of the ^13^C chemical shift using the GIAO method^35^ with the larger basis set 6–311+G(2d,p). All the calculations were performed in dichloroethane solvent using the conductor polarizable continuum model (CPCM).^36^,^37^ Two sets of calculations were made: one for the empty host and one with a chloroform molecule inside the cavity. The van der Waals interaction was taken into consideration in all structures optimized with the B3LYP functional by adding the empirical term to the DFT energies.^38^

## Results and Discussion

### NMR spectroscopy and DFT calculations of the free host

If we compare structures of the two diastereoisomers, cryptophane-C (*anti*) and cryptophane-D (*syn*), we notice that the two CTB rings are shifted relative to each other (see Scheme 1). This means that the methoxy groups of cryptophane-C (attached to one of the rings) move closer to the opposite CTB, thus causing the entrance gate for the guests into the cavity to become narrower than in cryptophane-D.

**Figure 1:**
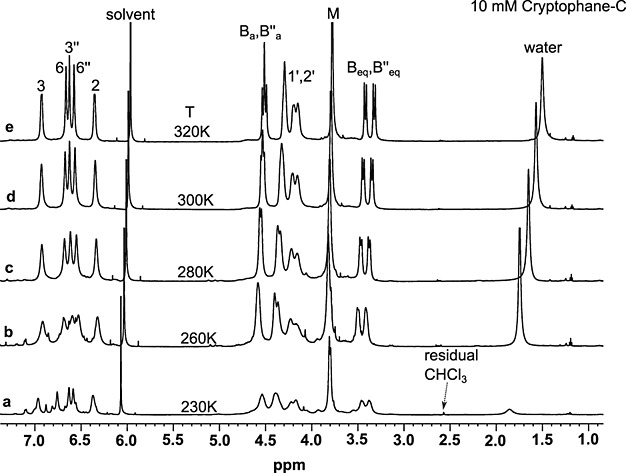
Variable temperature proton spectra for cryptophane-C in the absence of chloromethanes (10 mM, 14.1 T).

**Figure 2:**
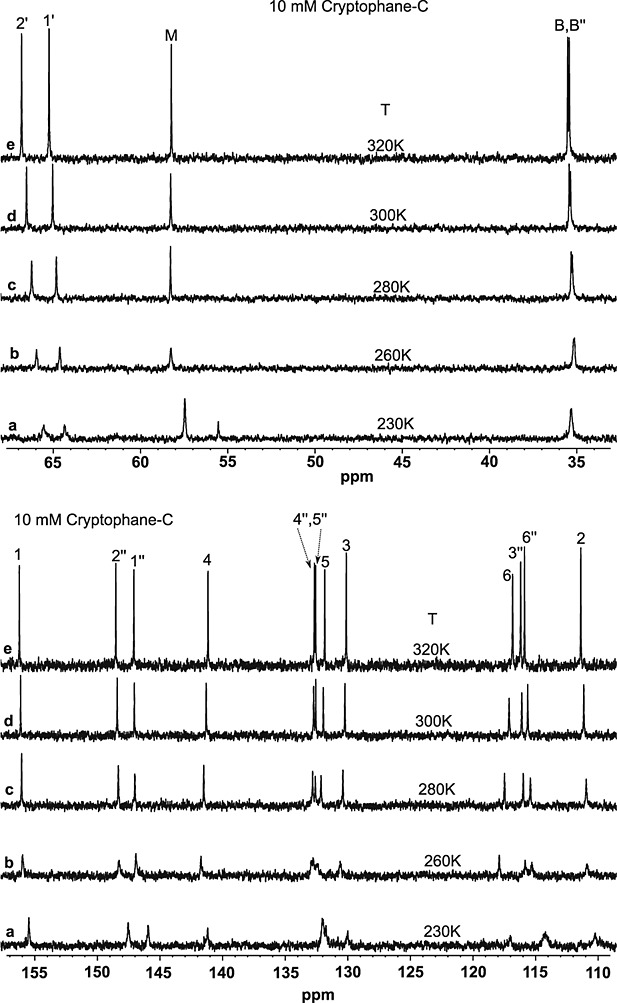
Variable temperature carbon-13 spectra for cryptophane-C in the absence of chloromethanes (10 mM, 14.1 T). (a) Aliphatic region; (b) aromatic region.

Variable temperature ^1^H and carbon-13 spectra of the “empty” host with the signal assignment are shown in Figs 1 and 2. The proton spectra at high temperature in Fig. 1 display quite narrow lines, whereas the spectra at lower temperatures broaden and split giving evidence of chemical (conformational) exchange. As shown in Fig. 1, the sample contains a certain amount of water, which probably enters the hydrophobic cavity,^39^ and chloroform originating from the synthesis is present in trace amount only. The exchange phenomena are also evident in the carbon-13 spectra (Fig. 2). In the carbon spectra, we are going to pay large attention to the signals of the linker carbon, C1′ and C2′, around 65 ppm. The measured chemical shift differences (δ2′–δ1′) between the linker carbons are much smaller than that in the case of cryptophane-D. The (δ2′–δ1′) reaches at most 2 ppm in the ^13^C spectra.

**Scheme 2:**
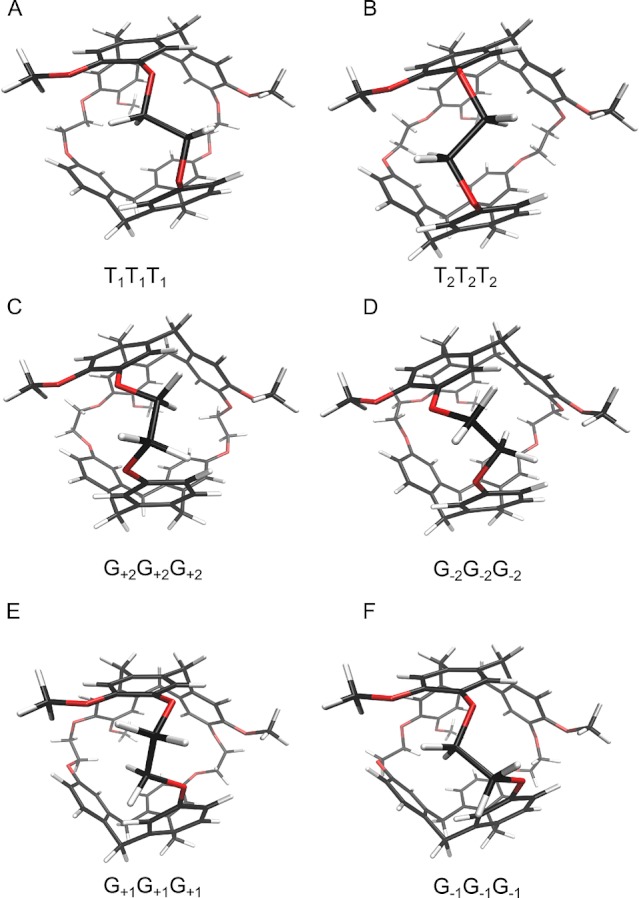
Selected conformers of cryptophane-C.

**Figure 3:**
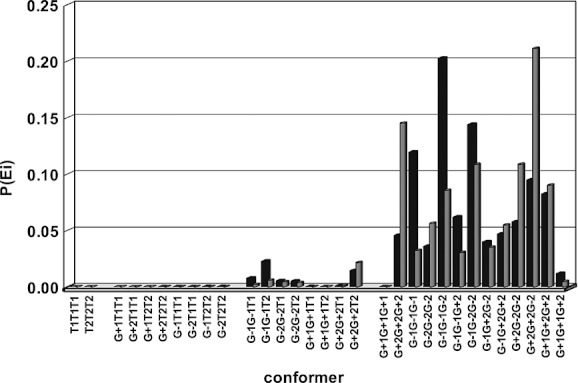
Calculated Boltzmann distribution of conformers for the guest-free host. The diagram is based on energies including the zero-point, thermal and van der Waals corrections. The black box correspond to the smaller basis set while the gray cylinders are from calculations using the larger basis set.

As in the case of all cryptophanes, the measured spectra are the result of averaging spectra of several conformers with different weights. The types of conformers of the linkers are the same as for cryptophane-D: *T*_1_, *T*_2_, *G*_+/−1_, *G*_+/−2_.^18^ Some of the interesting conformers of cryptophane-C are shown in Scheme 2. According to DFT calculations, the conformational distribution of the linkers is here very different from the case of the *syn* isomer (cryptophane-D), with the lowest energies corresponding to combinations of *gauche* arrangements. The full set of the energetics results is shown in the supplementary material, whereas the Boltzmann distribution of the conformers for the guest-free host is shown in Fig. 3. Clearly, the energetics and the population distribution are dependent on the basis set. Our larger basis set is not close to the saturation limit, and an even larger basis set would probably again affect the distribution. However, we wish to stress the main points that there are many energetically possible conformations.

**Figure 4:**
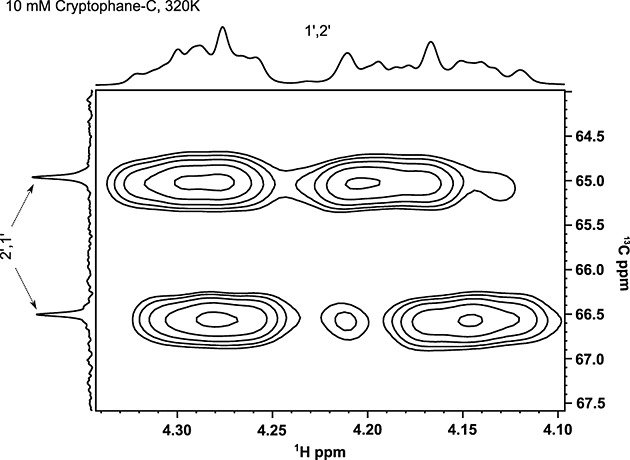
Part of an HSQC spectrum at 9.4 T, 10 mM of guest-free cryptophane-C at 320 K.

The DFT was also used to calculate the chemical shifts of the linker carbons, compare the supplementary material for the full set of data. Here, it suffices to say that the individual conformers are characterized by varying (δ2′–δ1′), but never higher than 5 ppm. Moreover, the observed increase of the linker chemical shift difference at higher temperature agrees with the increasing populations of the *G*_−1_ conformers. An interesting observation is that because of the small chemical shift difference, the HSQC measurements cannot give a safe assignment of peaks even at 320 K as it can be seen in Fig. 4. We shall return to the calculated chemical shift differences later on.

The cavity volumes of the most important conformers were estimated using the software Voidoo.^40^,^41^ The results are shown in Table 1.

**Table 1:** The estimated cavity volumes obtained using the software Voidoo^40^,^41^

Conformer	*V* (Å^3^)
*T*_1_*T*_1_*T*_1_	136
*T*_2_*T*_2_*T*_2_	106
*G*_*+*2_*T*_1_*T*_1_	130
*G*_−2_*T*_1_*T*_1_	114
*G*_−2_*G*_−2_*T*_2_	103
*G*_−2_*G*_−2_*T*_1_	102
*G*_*+*2_*G*_*+*2_*T*_2_	105
*G*_*+*2_*G*_*+*2_*T*_1_	114
*G*_*+*1_*G*_*+*1_*G*_*+*1_	97
*G*_−1_*G*_−1_*G*_−1_	79
*G*_*+*2_*G*_*+*2_*G*_*+*2_	104
*G*_−2_*G*_−2_*G*_−2_	88

The probe radius used for the volume estimation was 1.4 Å.

## Kinetics, Thermodynamics and Dynamics of Formation of Inclusion Complexes

### Dichloromethane

**Figure 5:**
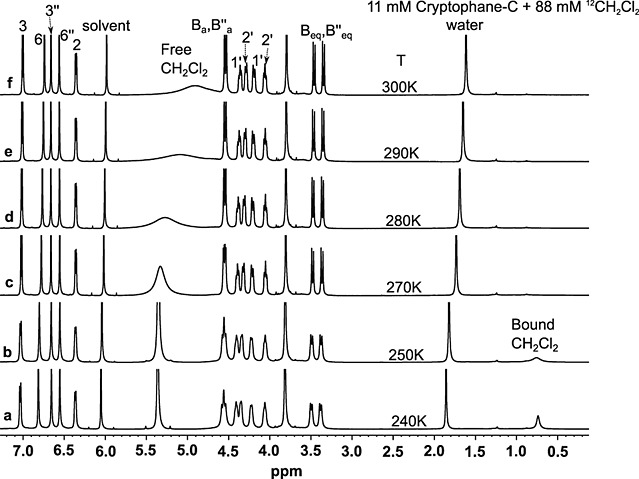
Variable temperature proton spectra for the sample of 11 mM cryptophane-C and 88 mM dichloromethane (14.1 T).

**Figure 6:**
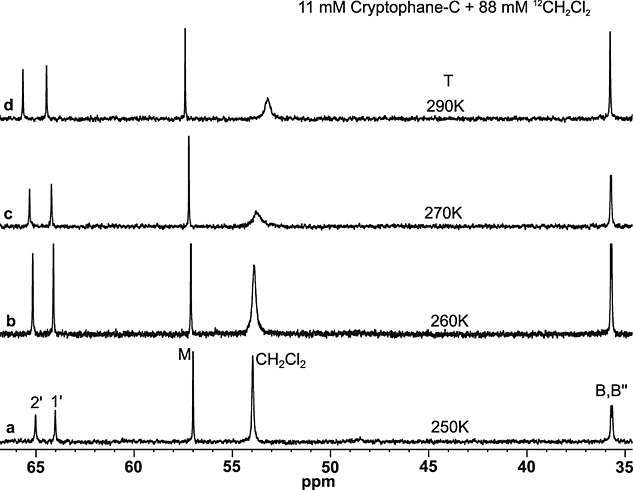
Variable temperature cabon-13 spectra (aliphatic region) for the sample of 11 mM cryptophane-C and 88 mM dichloromethane (14.1 T).

**Figure 7:**
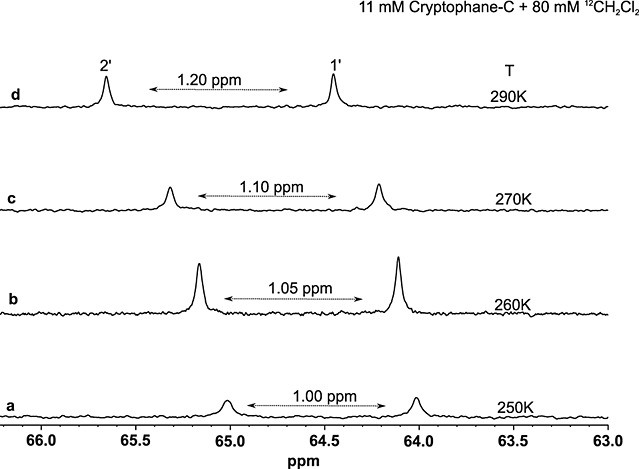
Variable temperature cabon-13 spectra (linker region) for the sample of 11 mM cryptophane-C and 88 mM dichloromethane (14.1 T).

The encapsulation of dichloromethane causes changes in the ^1^H and ^13^C spectra and their temperature dependence. Variable temperature ^1^H spectra from a sample with a large excess CH_2_Cl_2_ guest are shown in Fig. 5. At the lowest temperatures (240 and 250 K), we can see separate peaks for the free and bound guest, whereas at higher temperatures, the exchange between the two sites becomes faster. The conformational exchange of the host is changed by the binding of dichloromethane, which is most clearly seen on the aromatic and H1′ and H2′ peaks. The fact that the spectra acquired at lower temperature display a single set of well-resolved signals is an evidence that conformational exchange of the host is largely hindered by dichloromethane complexation. The VT ^13^C spectra in the aliphatic region are shown in Fig. 6. Also here, we can see less temperature-dependent effects compared with the free host. This is particularly clear in the case of the methoxy groups. The ^13^C spectra in the aromatic region (not shown) display little temperature dependence too. We zoom in on the linkers region in Fig. 7, where we can see that the individual chemical shifts, as well as (δ2′–δ1′) undergo significant changes with temperature.

**Figure 8:**
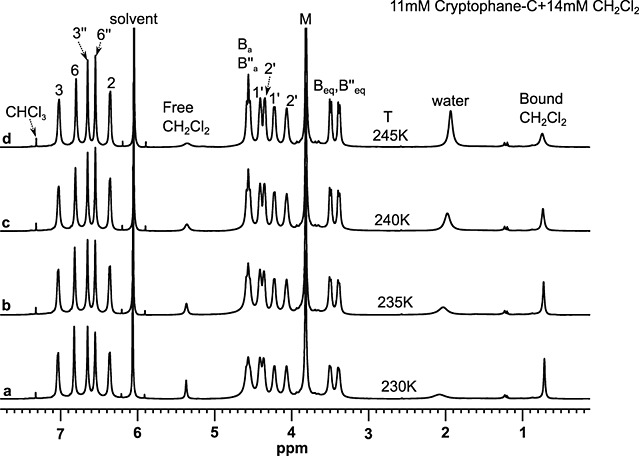
Variable temperature proton spectra for the sample of 11 mM cryptophane-C and 13.8 mM dichloromethane (14.1 T).

For the quantitative interpretation of the kinetics of the guest exchange between the free and the bound sites as well as of the equilibria of the complexation process, it is actually more advantageous to work with a sample with similar total concentrations of the guest and the host. The VT ^1^H spectra for such a sample are shown in Fig. 8. The exchange between the free and the bound sites of the host is in the fast regime throughout the whole investigated temperature range, whereas the proton signals of the free and bound guest at lower temperatures are broadened but well separated. Here, the ratio of free to bound guest and the dynamics of the guest exchange can be investigated by line-shape fitting. At the lowest temperatures, simple integration of the peaks is also possible. The results of the fitting are shown in Table 2. At temperatures higher than 245 K, the line-shape fitting is difficult because overlapping occurs and the faster exchange averages the peak position to the regions of the aliphatic peaks. The ratio of the obtained effective exchange rates is equal to the concentration ratio between the bound and the free guest, which could be confirmed by integration at 230 K.

**Table 2:** Line-shape fitting for the sample with cryptophane-C to dichloromethane ratio close to 1:1

Temperature (K)	*p*_f_	*p*_f_	*k*_fb_ (s^−1^)	*k*_bf_ (s^−1^)	*k*_fb_/*k*_bf_
230	0.308 (1)	0.692 (1)	36.8 (6)	16.4 (3)	2.24
235	0.320 (1)	0.680 (1)	65.8 (1)	31.0 (1)	2.12
240	0.323 (1)	0.677 (1)	116 (1)	55.2 (1)	2.09
245	0.338 (6)	0.662 (6)	226 (7)	116 (7)	1.96

Errors in the populations and rate constants (given in parentheses) were estimated using a 1000-step Monte Carlo procedure with a conservatively assumed 3% standard deviation in the point intensities.

We can see that the bound state is always more populated (approximately twice at 230–245 K) than the free state of CH_2_Cl_2_ guest. This observation immediately implies a high association constant. From the relative populations and the known total concentration of cryptophane-C (from weighing the dry sample after extraction with CCl_4_), we calculated concentrations of the different species (see Table 3). The symbol *k*_1_ = *k*_fb_ / [H] is the rate constant for the second-order reaction 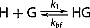. From the simple Arrhenius plot of the forward (*k*_1_) and backward (*k*_bf_) rate constants, we estimate the activation energies to 49 and 60 kJ/mol, respectively. The activation energy for the complexation reaction agrees with the value (46 kJ/mol) reported by Canceill *et al*.1985 The van't Hoff plot of the association constant reveals a low reaction enthalpy of −11 kJ/mol.

**Table 3:** Equilibrium concentrations in the solution with total concentrations [H]_tot_ =11 mM for the host, [G]_tot_ =[CH_2_Cl_2_]_tot_= 13.8 mM

Temperature (K)	[HG] (mM)	[G] (mM)	[H] (mM)	*k*_1_ (s^−1^M^−1^)	*K* (M^−1^)
230	9.5	4.2	1.5	24 700	1500
235	9.4	4.4	1.65	40 000	1300
240	9.3	4.5	1.7	68 000	1200
245	9.1	4.6	1.9	120 000	1000

### Chloroform

**Figure 9:**
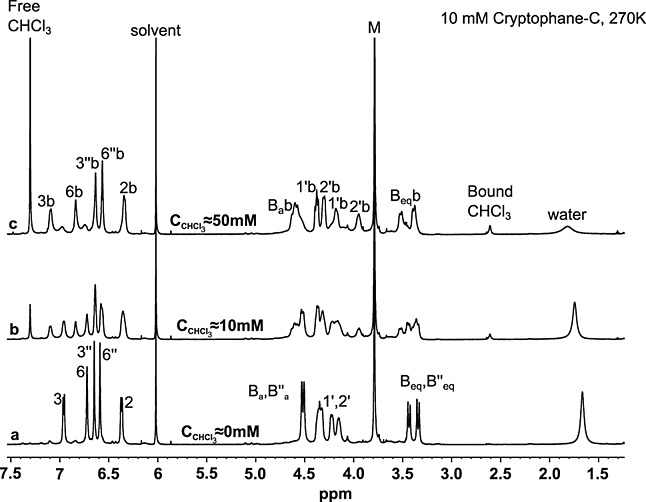
Proton spectra for the sample of 10 mM cryptophane-C with variable concentration of chloroform (270 K, 14.1 T).

**Figure 10:**
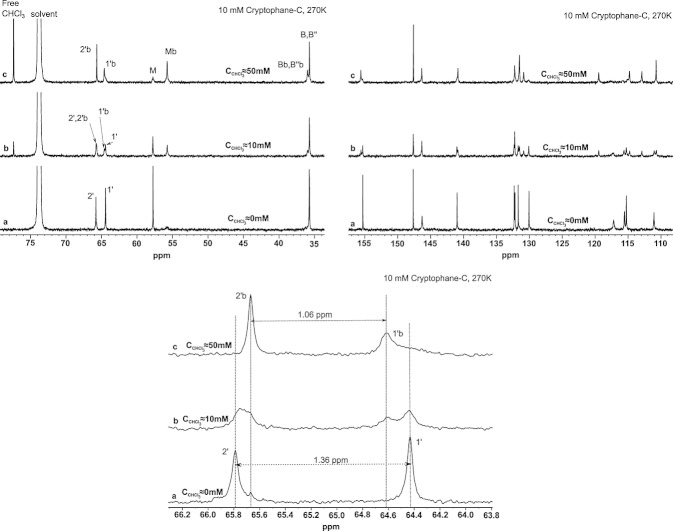
Carbon-13 spectra for the sample of 10 mM cryptophane-C with variable concentration of chloroform (270 K, 14.1 T). (a) Aliphatic region; (b) aromatic region; (c) the linker region.

The proton and carbon-13 spectra for the 10 mM cryptophane-C with 10 and 50 mM chloroform, measured at 270 K and 14.1 T, are shown in Figs 9 and 10. For comparison, the spectra of the guest-free sample of cryptophane-C are also included in Figs 9 and 10. Upon addition of chloroform, the host ^1^H signals in Fig. 9 broaden significantly, and some new lines appear, indicative of the exchange phenomena between the free and the bound host and possibly of the guest inclusion affecting the conformational equilibria of the host. The aromatic part of the ^13^C spectra (Fig. 10b) is even more informative. Most of the signals in the sample with 50 mM guest (where a large share of the host is filled with guest) are shifted somewhat with respect to the empty host. In the sample containing 10 mM CHCl_3_, many peaks are doubled: we see the resonances from the free and bound host in slow exchange. This observation is similar to our findings in the case of cryptophane-D,^18^ but the effects in the present work are less distinct. In Fig. 10c, we concentrate on the interesting region of the linker carbons. We can notice here that the chemical shift difference between the linker peaks is very close to the one in the case of dichloromethane guest.

**Figure 11:**
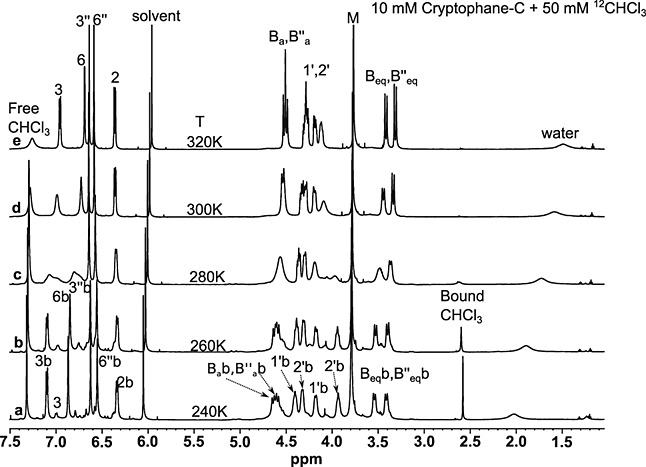
Variable temperature proton spectra for the sample of 10 mM cryptophane-C and 50 mM chloroform (14.1 T).

The variable temperature ^1^H spectra for the sample with 50 mM CHCl_3_ are shown in Fig. 11. At the two lowest temperatures (240 and 260 K), the chloroform signal is in slow exchange between the free and the bound sites, whereas the exchange process becomes faster at higher temperatures. In the temperature range 240–260 K, we were thus able to measure the bound-to-free exchange rates (*k*_bf_) using the exchange spectroscopy method. Here, we used the ^13^C-labeled sample to get rid of the inconveniently long ^1^H spin-lattice relaxation for free chloroform in the natural abundance material. The results are shown in Table 4. The free-to-bound rates, *k*_fb_, were obtained from *k*_bf_ and the equilibrium constant (see following paragraphs) using the principle of detailed balance. The rate constants are similar to values given in our earlier study,^17^ when one corrects for the temperature miscalibration in that study. The Arrhenius plot over the rather narrow temperature range gives rough estimates of the activation energies of approximately 80 kJ/mol for both the free-to-bound and bound-to-free reactions. This activation energy for the complexation reaction is significantly higher than that reported by Canceill *et al*.1985 The rate constant of backward reaction *k*_bf_ could be determined at limited number of temperature points only due to several factors that complicate the quantitative analysis. First, the ^1^*J*_CH_ coupling constant in ^13^CHCl_3_ is 210 Hz. One component of ^13^CHCl_3_ doublet is very close to the H3 peak of the bound host making the integration difficult. The line-shape fitting might be possible but has the disadvantage of many overlapping peaks and medium fast exchange of the free host peak. Summarizing, the spectra here are more complicated than in the case of cryptophane-D systems because the exchange situation (both host conformations and guest-host) is different. Nevertheless, the quantitative information, when possible to obtain, is very useful. The exchange rates are clearly much bigger here than in cryptophane-D, which seems to contradict the observation of the narrower gateways to the cavity in cryptophane-C. A possible explanation of this apparent contradiction is that the act of the guest entering or leaving the cavity does not occur by pressing the guest next to the methoxy group in a position shown in Scheme 1 but rather requires an “unlocking” of the gate through rotation of the methoxy group (see following paragraphs).

**Table 4:** Effective exchange rates for the free-to-bound and bound-to-free reactions in 10 mM cryptophane-C sample with 50 mM labeled CHCl_3_

Temperature (K)	*k*_fb_ (s^−1^)	*k*_bf_ (s^−1^)
240	0.06	0.47
246		1.29
250	0.47	3.4
254		5.0
258		8.2
260	1.34	10.7

Next, we turn to the determination of the equilibrium constant. This was carried out using three extraction-purified samples containing increasing amount of CHCl_3_ (see Materials and Sample Preparations section), making use of the integrated signal intensities for the three species (these can be best determined for the samples with low guest to host ratios). The results are shown in Table 5. It is interesting to compare the association equilibrium constants for cryptophane-C complex with the two chloromethanes. Clearly, the affinity of the host to dichloromethane is higher, in agreement with the findings by Canceill *et al*.1985

**Table 5:** Equilibrium constant for the reaction H + CHCl_3_ ⇄ CHCl_3_ @ H as a function of temperature

Temperature (K)	Association constant (M^−1^)
240	104
250	82
260	58
270	23

The temperature dependence of the equilibrium constant yields the reaction enthalpy of −26 kJ/mol and the entropy −67 J/(molK), leading to Δ*G* of approximately −8 kJ/mol at 260 K. We can notice that the sizable negative reaction enthalpy is not quite consistent with very similar values of the activation energies of the forward and backward reactions.^15^ The explanation of this inconsistency is probably to be sought in the differences in the sample compositions.

**Figure 12:**
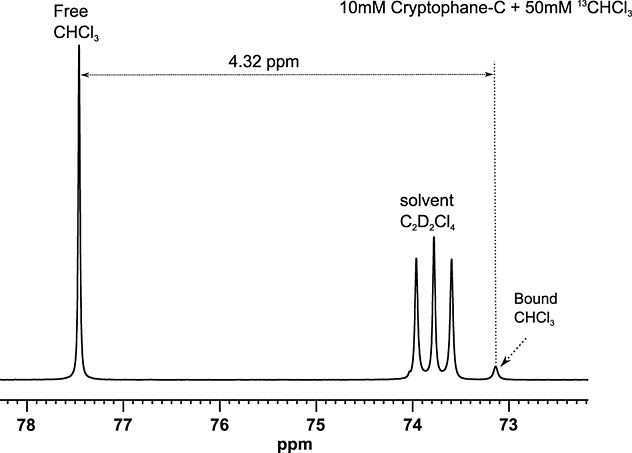
Carbon-13 signals for the sample of 50 mM labeled ^13^CHCl_3_ at 240 K and 14.1 T.

The labeled sample (10 mM cryptophane-C, 50 mM ^13^CHCl_3_) was also used in measurements of the guest carbon-13 spin-lattice relaxation rate and NOE. As discussed in earlier studies,^11^,^12^,^16^,^18^ the slow chemical exchange between the bound and the free positions renders the relaxation process biexponential and affects the measured NOEs of the two sites. We found the exchange conditions at 240 K suitable for relaxation measurements (cf. Fig. 12). Following the analysis protocol described in our earlier studies, we obtained the ^13^CHCl_3_ spin-lattice relaxation rate (*R*_1_) and the NOE parameters given in Table 6. The data for the free chloroform show full NOE and field-independent *R*_1_. To analyze the data in terms of a motional model, we estimated the global correlation time of the host at 240 K from the Arrhenius type of plot for various cryptophanes.^16^ It is approximately 4.4 ns. Using this value and the motional model introduced by Lipari and Szabo,^42^ we estimated the local correlation time (28 ±8 ps) and the generalized order parameter (0.68 ±0.11) for the bound chloroform. The order parameter is somewhat higher than in the case of CHCl_3_–cryptophane-D, indicating a more compact structure. If the local motion is treated as diffusion in a cone, the corresponding semi-angle is 29°.

**Table 6:** Carbon-13 relaxation data at 240 K and at two magnetic fields for ^13^CHCl_3_, free and bound in the cryptophane-C cavity

Site	*R*_1_ (14.1 T) (s^−1^)	NOE (14.1 T)	*R*_1_ (16.5 T) (s^−1^)	NOE (16.5 T)
Free	0.28 (0.003)	3.01 (0.41)	0.28 (0.02)	2.8 (0.4)
Bound	1.47 (0.03)	1.47 (0.19)	1.07 (0.03)	1.15 (0.1)

## Structure of CHCl_3_ and CH_2_Cl_2_ complexes with cryptophane-C

### DFT calculations and chemical shifts

Extensive DFT calculations were carried out for the host complexed with CHCl_3_, allowing the CH bond in chloroform to point toward each of the CTB units. Similarly to the case of cryptophane-D and to the free cryptophane-C host, several conformers can be populated in the CHCl_3_–cryptophane-C. Table of energies calculated on different levels of theory is shown in the supplementary material. They differ significantly from the values given in our earlier work.^17^ The geometry optimization in that earlier work was not fully converged.

**Figure 13:**
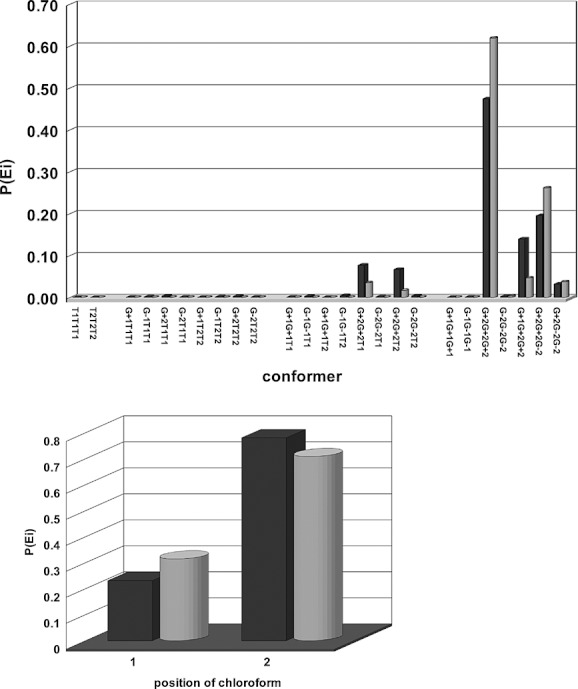
Calculated Boltzmann distribution of selected conformers for the chloroform-containing host. (a) Distribution of conformers summed over the two chloroform orientations. (b) Distribution of species with two different orientations of chloroform guest summed over the linker conformations. Orientation 1 refers to the chloroform CH bond pointing toward methoxylated cap, whereas orientation 2 refers to the opposite case. The diagram is based on energies including the zero-point, thermal and van der Waals corrections. The black boxes correspond to the smaller basis set while the gray cylinders are from calculations using the larger basis set.

The Boltzmann distribution converted to equilibrium populations of conformers of cryptophane-C summed over both the low-energy orientations of CHCl_3_ is shown in Fig. 13a. The conformational space is found considerably restricted compared with the guest-free cryptophane-C. The conformations populated contain mainly *G*_+2_ and *G*_−2_ geometries of individual linkers. Conversely, Fig. 13b shows the corresponding relative populations of the species that differ by orientation of chloroform guest, summed over conformations of linkers.

Besides the relative energies of the conformers, the calculated chemical shifts of the linker carbons are very informative, in analogy with what we showed earlier in the case of cryptophane-D systems.^18^ The chemical shift differences between the chemical shifts of the linker carbons C1′ and C2′ (δ2′–δ1′), for some of the conformers, both in the free form of cryptophane-C and in the complex with chloroform, are listed in Table 7. For the complete set of the differences, please consult the supporting material. The difference is found much smaller than in the case of the complex of cryptophane-D.

**Table 7:** Calculated differences between chemical shifts for carbons 1′ and 2′ in the guest-free and CHCl_3_-containing host

conformer	(δ2′–δ1′) (ppm), free host	(δ2′–δ1′) (ppm), CHC1_3_ in orientation 1	(δ2′–δ1′) (ppm), CHC1_3_ in orientation 2
*T*_1_*T*_1_*T*_1_	0.2	-1.5	0.4
*T*_2_*T*_2_*T*_2_	0.7	-0.4	0.8
*G*_+1_*G*_+1_*G*_+1_	2.3	2.3	2.7
*G*_−1_*G*_−1_*G*_−1_	4.4	4.1	4.0
*G*_+2_*G*_+2_*G*_+2_	2.1	2.8	2.5
*G*_−2_*G*_−2_*G*_−2_	-1.7	-1.9	-1.6

Orientation 1 refers the CH bond in chloroform pointing toward the methoxylated cap, whereas the CH bond points in the opposite direction in orientation 2.

As it was mentioned earlier, it is not possible to uniquely assign the peaks in the guest-free case. Moreover, the chemical shift difference (δ2′–δ1′) is 1.5 ppm at 320 K and decreases when the sample is cooled down. We can also notice that the spectra are slightly shifted downfield. In the case of cryptophane-D, there was no field shift nor any chemical shift difference change. The changes between the complexed and free host are very similar, meaning that the effect of the guest is much less pronounced than in the case of cryptophane-D. The Boltzmann-weighted average of the calculated chemical shift differences at 298 K are approximately 1.5–2 ppm (depending on the method to get the relative energies), both for the free and CHCl_3_-loaded host. The measured value is 1.47 ppm for the free host and 1.38 ppm for the CHCl_3_ complexed host; at 290 K, the (δ2′–δ1′) value for the CH_2_Cl_2_ is 1.2 ppm. Thus, we can see that both the measurements and calculations predict that the structures of the complexes are very close to each other.

### Cross relaxation of host protons

The most important result of our previous study of the CHCl_3_–cryptophane-C system was that one of the guest orientations (orientation 2) was significantly more probable than the other one. This conclusion based on NOESY and ROESY experiments was fully confirmed in this study. Here, we go deeper in the cross-relaxation measurements and concentrate on dipole–dipole interactions between pairs of host protons, measured using 2D NOESY and ROESY. From the cross-relaxation rates, it is possible to obtain information on the conformational equilibria of the linkers between the two caps. The cross relaxation between a pair of protons is a very sensitive measure of distance because the rate constant includes the dipolar coupling constant squared in the expression.^43^ The square of the strength of dipolar interaction decays as *r*^−6^, where *r* is the distance between the two protons in question. Thus, a distance change from 2 to 4 Å results in the cross-relaxation rate, which is 64 times smaller. The distances between the linker protons and the aromatic protons in the cryptophane-C molecule fall within the range of 2 and 6 Å. Thus, we analyzed both the 2D NOESY and ROESY spectra in the initial rate approximation (the longest mixing time used was 240 ms in NOESY and 200 ms in ROESY) where the cross relaxation is linear in the mixing time.^27^,^28^ The measurements were time consuming because of the long (5–7 s) relaxation delays and the high number of scans needed (eight scans in the direct domain and 512–1024 increments in the indirect dimension).

**Figure 14:**
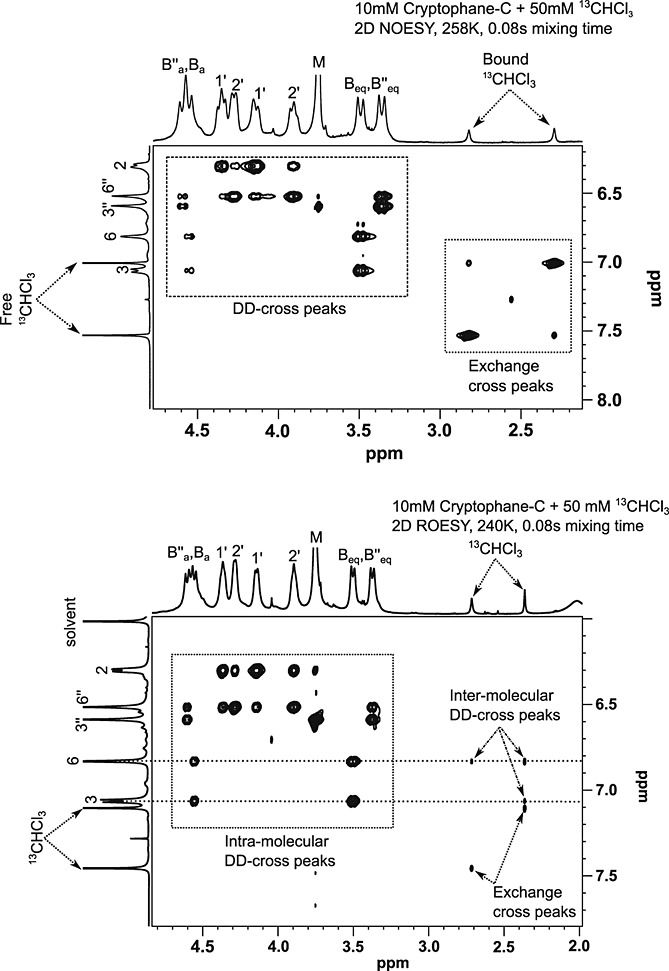
Intrahost cross-relaxation spectra. (a) NOESY for 50 mM labeled ^13^CHCl_3_, 9.4 T, 258 K, mixing time 0.08 s. (b) ROESY for 50 mM labeled ^13^CHCl_3_, 14.1 T, 240 K, *B*_1_ = 3.0 kHz, mixing time 0.08 s.

Examples of 2D NOESY and ROESY spectra are shown in Fig. 14. An interesting observation, particularly clear in the projection of the ROESY spectrum, is that the signal heights (and widths) for the two lines in the *J*-coupled doublet of ^13^CHCl_3_ are not equal. This is most probably an effect of the interference of the dipole–dipole and chemical shielding relaxation mechanisms (cross-correlated relaxation).^44^ The unequal intensity pattern is also seen in the 2D ROESY spectrum in the form of a “missing” intermolecular cross peak with intensity just below the threshold used when printing the spectrum.

In the spectra of both complexes (CH_2_Cl_2_–cryptophane-C and CHCl_3_–cryptophane-C) at 258 K, the peaks of the linker protons are sufficiently separated to allow integration of the diagonal and cross peaks between the linker protons (1′,2′) and aromatic protons (2,3,6,3″,6″). Actually, there are two nonequivalent protons, denoted 1′a and 1′b, bound to carbon 1′, and two nonequivalent protons, 2′a and 2′b, bonded to carbon 2′.

Generally, the signal-to-noise ratio is lower in the case of ROESY; thus, the error is higher in the evaluated integrals. Nevertheless, the information content in the NOESY and ROESY data is essentially the same. The measured cross-relaxation rates from the variable mixing time experiments are summarized in Table 8. The measured values are relatively high compared with the cross-relaxation rates between the guest proton and the aromatic protons.^17^ The distances between the linker protons and the aromatic protons, taken from the DFT-optimized structures, are listed in Table 9 for a comparison.

**Table 8:** Intrahost cross-relaxation rates in CHCl_3_–cryptophane-C

^1^H	2		3		6		3″		6″	
	NOE	ROE	NOE	ROE	NOE	ROE	NOE	ROE	NOE	ROE
1′a	0.46	0.48	0.03	—	0.06	0.06	—	—	0.13	0.27
1′b	0.88	2.18	0.05	—	0.07	0.06	—	—	0.26	0.58
2′a	0.15	0.29	0.01	—	0.04	0.04	—	—	0.46	0.86
2′b	0.3	0.83	0.02	—	0.05	0.03	—	—	0.82	1.25
M	—	—	—	—	—	—	0.12	0.27	—	—

Rates are listed between the linker and methoxy protons on the one hand and the aromatic protons on the other hand. All rates in s^−1^.

**Table 9:** Intrahost distances (Å) in the CHCl_3_ complexed conformers of cryptophane-C, obtained from the DFT-optimized structures

^1^H	**2**	**3**	**6**	**3**″	**6**″	**2**	**3**	**6**	**3**″	**6**″

*G*_−2_*G*_−2_*G*_−2_(2)						*G*_+2_*G*_+2_*G*_+2_(2)				
**1′a**	2.55	4.52	4.15	3.65	2.57	2.1	4.51	4.48	6.01	4.61
**1′b**	2.28	4.72	4.60	4.58	3.60	3.68	5.98	4.38	6.66	4.67
**2′a**	4.51	6.82	5.70	4.19	2.18	2.70	4.53	4.56	3.95	2.44
**2′b**	4.57	6.86	4.78	5.78	3.71	3.53	5.78	5.72	4.26	2.47
*T*_1_*T*_1_*T*_1_(2)						*T*_2_*T*_2_*T*_2_(2)				
**1′a**	2.65	4.57	3.99	4.69	3.29	4.04	5.56	3.55	4.91	4.66
**1′b**	2.55	4.97	4.61	5.37	3.86	4.43	6.44	3.55	7.14	5.07
**2′a**	4.78	6.67	6.32	4.51	4.24	3.03	4.87	4.57	4.19	2.73
**2′b**	4.76	6.40	4.71	5.87	4.46	3.37	5.72	5.04	5.31	3.52
*G*_−1_*G*_−1_*G*_−1_(2)						*G*_+1_*G*_+1_*G*_+1_(2)				
**1′a**	4.37	5.76	3.70	6.61	4.56	4.58	4.32	2.45	6.00	5.59
**1′b**	4.46	4.20	2.17	7.50	5.50	3.80	5.76	2.40	4.60	4.44
**2′a**	4.55	3.66	2.52	4.92	4.59	4.55	6.84	4.66	6.17	4.27
**2′b**	4.55	3.66	2.52	4.65	3.90	5.70	6.00	4.54	4.83	4.52

All the data are for orientation 2 of chloroform.

Let us in particular consider the large cross-relaxation rates for protons 1′a and 1′b with proton 2 and for protons 2′a and 2′b with protons 6″ (light-blue background in Table 8). It can be seen from the two tables that the measured cross-relaxation rates are only consistent with the linkers spending most of their time in the *G*_+2_ and *G*_−2_ conformation, since the remaining conformations cannot produce large cross-relaxation rates. Moreover, the change in the dihedral angle from −67º (*G*_−2_) to +67º (*G*_+2_) imposes a different effect on each proton within a diastereomeric pair: 1′a remains at close distance to 2 while 1′b gets much further from 2 (3.68 Å). Similarly, 2′a remains close to 6″ while the initially distant 2′b (3.71 Å) gets much closer (for details, see Table 9). The explanation of the relatively large and mutually comparable cross-relaxation constants of the respective proton pairs (Table 8) thus requires the presence of both *G*_−2_*G*_−2_*G*_−2_ and *G*_+2_*G*_+2_*G*_+2_ conformers exchanging with each other. The ROESY/NOESY data are also important by telling us which conformations are not present in appreciable amount. For example, the slow cross relaxation of proton 6 excludes the large populations of *T*_2_*T*_2_*T*_2_ conformer as well as *G*_±1_*G*_±1_*G*_±1_. It may also be worthwhile to notice that similar conclusions concerning the dominance of *gauche* conformers were reached by Luhmer *et al*.1999 in their study of xenon binding in cryptophane-A cavity.

The combination of the experimental chemical shifts, NOESY/ROESY measurements and DFT calculations reveals that the accessible conformational space of cryptophane-C linkers becomes significantly restricted by inclusion of chloroform guest. Nevertheless, the linkers dynamics is not stopped but there is conformational exchange active among the populated conformations.

The cross-relaxation rate of the methoxy protons (Table 8) with the closest aromatic proton (3″) cannot be explained with the lowest energy structure from the DFT calculations, where the −CH-C-O-CH_3_ dihedral angle is 2.5°. In this configuration, the average distance of the methoxy protons to the 3″ is 2.7 Å. Thus, one would expect a much higher cross-relaxation rate. If one also takes into account the higher energy configuration (where the dihedral angle is around −110°, that is, the methoxy group points out of the aromatic plane opening up the “door” for CHCl_3_ or for CH_2_Cl_2_ to enter the cavity), then the distance from the aromatic proton 3″ increases to an average roughly 4 Å. From the cross-relaxation measurements and from the ^13^C spectra we can thus conclude that the methoxy group is indeed in exchange between the two positions. We believe that this is the origin of the kinetic barrier for the complexation reaction. Similar effects were also seen in the case of the cryptophane-D complex.^18^ It should be also noted that the cross-relaxation rates of the methoxy group are averages of the free and bound form because the corresponding peaks in the ^1^H proton spectra are not sufficiently separated in the case of cryptophane-C.

Very similar cross-relaxation rates between the linkers and aromatic protons were also obtained for the CH_2_Cl_2_–cryptophane-C complex. The data are shown in Table 10. The very low cross-relaxation rate of the methoxy group proton with the aromatic proton 3″ in the case of the CH_2_Cl_2_ complex (compared with the CHCl_3_ case) is surprising and may indicate that the methoxy group conformations with the dihedral angle close to −110° are even more abundant here. This would agree with fact that the exchange between the bound and free host is much faster for the CH_2_Cl_2_ guest than for CHCl_3_.

**Table 10:** Intrahost NOE cross-relaxation rates in CH_2_Cl_2_–cryptophane-C. Rates are listed between the linker and methoxy protons on the one hand and the aromatic protons on the other hand

^1^H	2	3	6	3″	6″
1′a	0.42	0.04	0.06	-	0.1
1′b	0.81	0.05	0.05	0.07	0.22
2′a	0.13	0.01	0.03	0.07	0.46
2′b	0.25	0.02	0.02	0.09	0.59
M				0.01	

All rates in s^−1^.

In fact, we propose the following mechanism for the guest complexation reaction. Not every collision between the host and the guest leads to successful reaction. In the case of CHCl_3_, two conditions should be fulfilled. First, the collision should happen when the methoxy groups point out of the plane of the ring, and second, the chloroform is oriented with the proton pointing toward one of the CTB rings. The second condition follows from size comparison between the opening and the guest. For dichloromethane, only the first condition needs to be met. Thus, we see the complexation process as a double conformational selection, where the “unlocked” methoxy group conformation is selected in the rate determining step. Once the guest is inside the cavity, the second selection is between possible conformations of the linkers.

The measured cross-relaxation rates show that the cryptophane-C behavior is different from its *syn* isomer, cryptophane-D. Here, the complex formation results in very similar structures in the case of both chloromethanes. Nevertheless, the complexation is still connected to a conformational selection (the second step in the previously mentioned model), as it can be seen in all the ^1^H and ^13^C spectra. In the case of the *anti* isomer, cryptophane-C, the main conformers correspond to the *gauche* 2 positions of the linkers. It is also noteworthy that, in the case of cryptophane-C complexes, the ^13^C chemical shifts of the linkers are less informative because the differences between individual conformers are much smaller than in the case of cryptophane-D. Moreover, the separation and assignment of the peaks is only possible when cryptophane-C is complexed with a chloromethane (see DFT calculations and ^13^C spectra). This also suggests that the noncomplexed host displays a large conformational flexibility, probably including also some conformers not accounted for in our DFT calculations. We notice that the ^13^C chemical shift difference between the two linker carbons (δ2′–δ1′) increases with increasing temperature. When temperature increases, the conformational and guest exchange processes are getting faster and the distributions of conformers are changed too.

## Conclusions

In conclusion, we have presented an extensive NMR investigation of cryptophane-C and its complexes with dichloromethane and chloroform. Combining variable concentration and variable temperature proton and carbon-13 spectra, host–guest and host–host cross-relaxation measurements and carbon-13 relaxation data provides a very detailed picture of interaction and dynamics. The NMR results are nicely corroborated by DFT calculations of energetics and carbon-13 chemical shifts. The association constant for dichloromethane with cryptophane-C is much larger than that for the chloroform. We believe that the thermodynamic stability of the complexes is related to the fact that the smaller dichloromethane guest fits better inside the host cavity. The complexation of chloromethanes into cryptophane-C and cryptophane-D cavities shows big differences between the two diastereomeric hosts. As opposed to cryptophane-D (*syn* isomer), cryptophane-C in the complexed form prefers the *gauche*+2 and *gauche*−2 conformations. In some other aspects, the two hosts show also a similar behavior, for example, concerning the limited reorientational freedom of chloroform as guest inside the host cavity.

The kinetics of the guest exchange between the free site in solution and bound site inside the cryptophane-C cavity differ also significantly between dichloromethane and chloroform: dichloromethane exchanges much faster than CHCl_3_. A plausible explanation of these findings is that the barrier related to the guest entering and leaving the cavity is higher for the bigger chloroform guest, which we believe is related to rotation of the methoxy groups.
