# Supplementary material for: Host–guest complexes between cryptophane-C and chloromethanes revisited
Source: Magn Reson Chem. 2012 Nov 7;51(1):19–31. doi: 10.1002/mrc.3898 (PMC3568900; doi:10.1002/mrc.3898)
Supplement: Supplementary file 1 [file mrc0051-0019-SD1.pdf]

# Supplementary Material to the Manuscript: Host-guest complexes between cryptophane-C and chloromethanes revisited

Z. Takacs[a], M. Šoltésová[a,b], J. Kowalewski[a], J. Lang[b], T. Brotin[c] and J.-P. Dutasta[c]

[a]*Department of Materials and Environmental Chemistry, Arrhenius Laboratory, Stockholm University, SE-10691 Stockholm, Sweden*
[b]*Department of Low Temperature Physics, Faculty of Mathematics and Physics, Charles University in Prague, V Holesovickach 2, CZ-180 00 Prague 8, Czech Republic*
[c]*Laboratoire de Chimie, CNRS-UMR 5182, Ecole Normale Superieure de Lyon, 46 Allée d'Italie, F-69364 Lyon cedex 07, France*

July 5, 2012

Table 1: The relative energies of non-complexed Cryptophane-C in Kj/mol. Lower case 'e' denotes the energies obtained by the basis set 6-31G(d), and upper case E denotes the energies obtained by single point calculations at the basis set 6-311+G(2d,p). 'EE' and 'ee' contain the zero point corrections and the thermal corrections. 'EEE' and 'eee' contain the van der Waals(vdW) correction too. The column 'vdW' is the absolute value of the van der Waals correction in Kj/mol. The abbreviation 'deg' is the degeneracy of the conformer used for the Boltzmann distribution. The conformers labeled 'b' were optimized with different methoxy group dihedral (3"-2"-O-M) see Table 2.

| Conformer/Energy | deg | e | E | ee | EE | eee | EEE | vdW |
|---|---|---|---|---|---|---|---|---|
| $T_1T_1T_1$ | 1 | 33.1 | 38.7 | 21.8 | 27.5 | 35.1 | 38.3 | -507.9 |
| $T_2T_2T_2$ | 1 | 31.5 | 36.1 | 23.2 | 27.8 | 26.5 | 28.7 | -518.0 |
| $G_{+1}T_1T_1$ | 3 | 24.8 | 30.9 | 21.4 | 27.6 | 27.0 | 30.7 | -515.6 |
| $G_{+2}T_1T_1$ | 3 | 23.4 | 27.4 | 15.2 | 19.1 | 25.0 | 26.6 | -511.3 |
| $G_{+1}T_2T_2$ | 3 | 29.7 | 38.0 | 21.3 | 29.7 | 29.1 | 35.1 | -513.4 |
| $G_{+2}T_2T_2$ | 3 | 22.7 | 25.7 | 16.7 | 19.7 | 23.6 | 24.2 | -514.3 |
| $G_{-1}T_1T_1$ | 3 | 21.7 | 27.7 | 18.1 | 24.0 | 19.8 | 23.4 | -519.5 |
| $G_{-2}T_1T_1$ | 3 | 22.6 | 27.4 | 18.1 | 22.9 | 22.3 | 24.7 | -517.0 |
| $G_{-1}T_2T_2$ | 3 | 26.5 | 32.9 | 18.1 | 24.4 | 18.9 | 22.9 | -520.4 |
| $G_{-2}T_2T_2$ | 3 | 22.9 | 26.9 | 18.4 | 22.5 | 18.9 | 20.5 | -520.8 |
| $G_{-1}G_{-1}T_1$ | 3 | 18.6 | 24.8 | 15.0 | 21.2 | 9.5 | 13.2 | -526.7 |
| $G_{-1}G_{-1}T_2$ | 3 | 17.5 | 23.7 | 15.5 | 21.7 | 6.8 | 10.6 | -529.9 |
| $G_{-2}G_{-2}T_1$ | 3 | 15.4 | 18.8 | 12.1 | 15.5 | 10.3 | 11.4 | -522.9 |
| $G_{-2}G_{-2}T_2$ | 3 | 17.4 | 21.1 | 11.2 | 15.0 | 10.5 | 11.8 | -522.0 |
| $G_{+1}G_{+1}T_1$ | 3 | 21.7 | 28.6 | 17.9 | 24.8 | 19.1 | 23.6 | -520.0 |
| $G_{+1}G_{+1}T_2$ | 3 | 24.0 | 32.0 | 19.6 | 27.7 | 21.8 | 27.4 | -519.0 |
| $G_{+2}G_{+2}T_1$ | 3 | 12.4 | 14.5 | 9.0 | 11.2 | 14.9 | 14.7 | -515.3 |
| $G_{+2}G_{+2}T_2$ | 3 | 10.1 | 12.0 | 7.3 | 9.2 | 8.0 | 7.5 | -520.5 |
| $G_{+1}G_{+1}G_{+1}$ | 1 | 19.4 | 27.0 | 20.1 | 27.7 | 17.1 | 22.3 | -524.1 |
| $G_{+2}G_{+2}G_{+2}$ | 1 | 0.0 | 0.0 | 0.0 | 0.0 | 2.4 | 0.0 | -518.8 |
| $G_{-1}G_{-1}G_{-1}$ | 1 | 11.4 | 17.5 | 13.9 | 20.0 | 0.0 | 3.7 | -535.1 |
| $G_{-2}G_{-2}G_{-2}$ | 1 | 8.6 | 10.3 | 11.2 | 12.9 | 3.0 | 2.4 | -529.4 |
| $G_{-1}G_{-1}G_{-2}$ | 3 | 9.2 | 14.2 | 13.2 | 18.2 | 1.4 | 4.0 | -533.0 |
| $G_{-1}G_{-1}G_{+2}$ | 3 | 10.3 | 14.9 | 12.6 | 17.2 | 4.4 | 6.6 | -529.4 |
| $G_{-1}G_{-2}G_{-2}$ | 3 | 8.2 | 11.8 | 12.5 | 16.0 | 2.3 | 3.4 | -531.4 |
| $G_{-1}G_{+2}G_{-2}$ | 3 | 9.7 | 12.9 | 11.5 | 14.7 | 5.4 | 6.2 | -527.2 |
| $G_{-1}G_{+2}G_{+2}$ | 3 | 6.0 | 8.5 | 8.2 | 10.7 | 5.0 | 5.1 | -524.4 |
| $G_{+2}G_{-2}G_{-2}$ | 3 | 5.7 | 7.0 | 10.4 | 11.7 | 4.5 | 3.4 | -527.1 |
| $G_{+2}G_{+2}G_{-2}$ | 3 | 2.7 | 3.6 | 6.0 | 6.8 | 3.3 | 1.8 | -523.8 |
| $G_{+1}G_{+2}G_{+2}$ | 3 | 2.9 | 5.5 | 5.4 | 8.0 | 3.6 | 3.9 | -522.9 |
| $G_{+1}G_{+1}G_{+2}$ | 3 | 10.0 | 15.2 | 11.4 | 16.5 | 8.5 | 11.2 | -524.1 |
| $G_{-1}G_{-1}G_{-1}b$ | 1 | 32.8 | 41.2 | 26.7 | 35.1 | 9.9 | 15.9 | -538.0 |
| $G_{-2}G_{-2}G_{-2}b$ | 1 | 27.3 | 30.0 | 20.6 | 23.3 | 17.0 | 17.4 | -524.7 |
| $G_{+2}G_{+2}G_{+2}b$ | 1 | 19.8 | 20.7 | 14.8 | 15.7 | 20.6 | 19.1 | -515.4 |
| $G_{-1}G_{+2}G_{-2}b$ | 3 | 16.6 | 19.8 | 14.7 | 17.9 | 10.3 | 11.0 | -525.6 |
| $G_{+1}G_{+2}G_{+2}b$ | 3 | 10.4 | 13.4 | 7.8 | 10.8 | 7.4 | 8.0 | -521.6 |

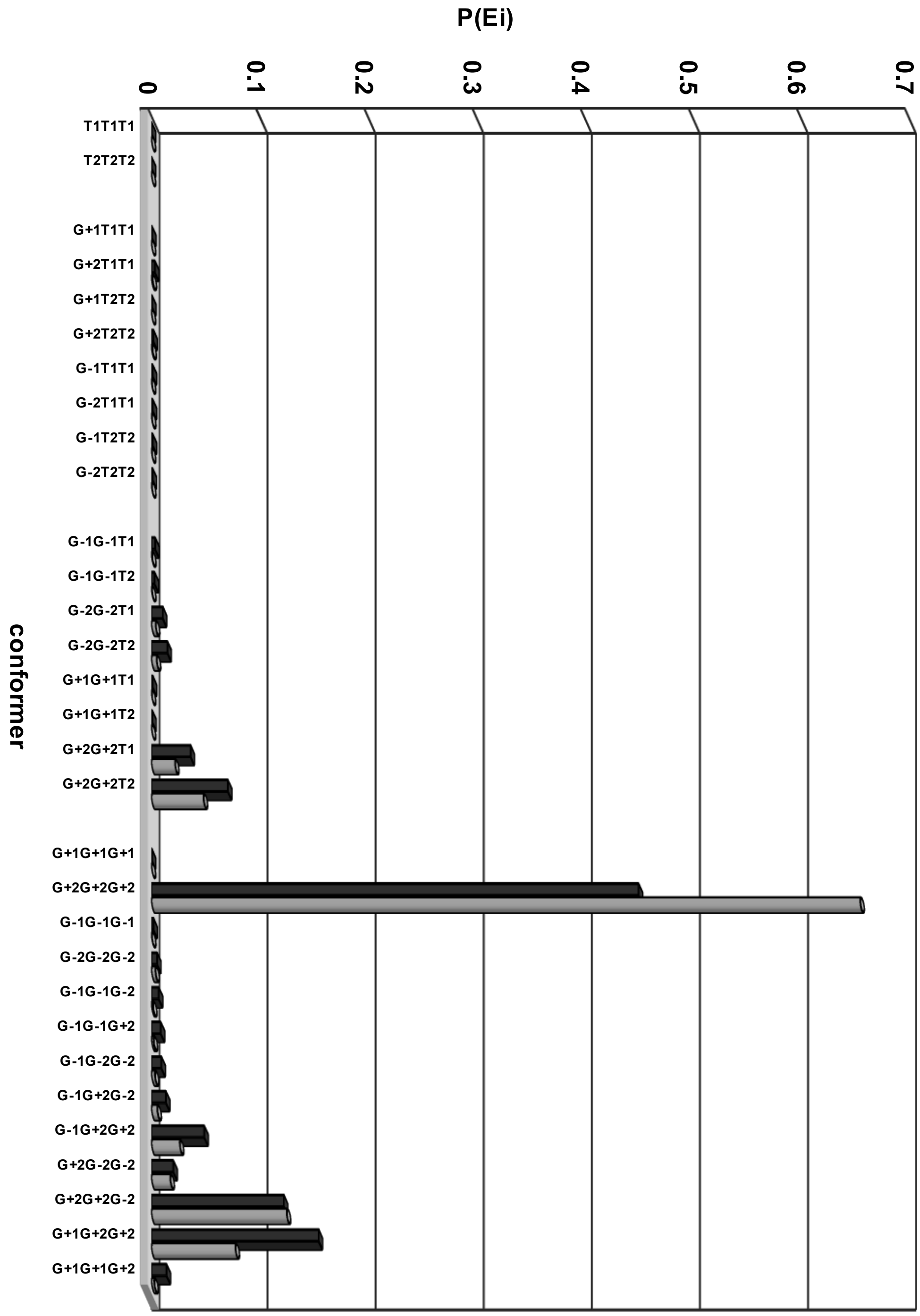


Figure S1: The Boltzmann distribution of conformers of non-complexed cryptophane-C based on the energies in column ee(box) and EE(cylinder) in Table 1.

Table 2: The dihedral angle of the linkers and methoxy groups of non-complexed Cryptophane-C. The conformers labeled by 'b' have different dihedral angle of the methoxy groups.

| Conformer/Dihedral | O-1'-2'-O | O-1'-2'-O | O-1'-2'-O | 3"-2"-O-M | 3"-2"-O-M | 3"-2"-O-M |
|---|---|---|---|---|---|---|
| $T_1T_1T_1$ | 177.2 | 176.9 | 177.0 | 2.8 | 3.0 | 2.4 |
| $T_2T_2T_2$ | -177.4 | 166.6 | 166.0 | 0.2 | 1.7 | 1.8 |
| $G_{+1}T_1T_1$ | 60.8 | -177.5 | 176.5 | 2.0 | 2.1 | 3.0 |
| $G_{+2}T_1T_1$ | 59.5 | 178.5 | 179.3 | 2.1 | 2.4 | 0.6 |
| $G_{+1}T_2T_2$ | 60.1 | -171.9 | -175.9 | 0.3 | 1.6 | 1.0 |
| $G_{+2}T_2T_2$ | 56.4 | 175.4 | 172.0 | 0.6 | 0.8 | 2.0 |
| $G_{-1}T_1T_1$ | -70.1 | 178.1 | -167.4 | 3.2 | 3.3 | 1.0 |
| $G_{-2}T_1T_1$ | -56.0 | 175.6 | -179.3 | 3.0 | -0.5 | 1.1 |
| $G_{-1}T_2T_2$ | -68.2 | 168.7 | -179.7 | 3.6 | 0.7 | 1.6 |
| $G_{-2}T_2T_2$ | -56.3 | -175.8 | 165.5 | 0.6 | 1.5 | -4.4 |
| $G_{-1}G_{-1}T_1$ | -68.1 | -68.4 | 179.2 | 2.9 | 1.5 | 1.4 |
| $G_{-1}G_{-1}T_2$ | -68.9 | -66.3 | 167.4 | 4.6 | 2.4 | 1.1 |
| $G_{-2}G_{-2}T_1$ | -56.1 | -58.2 | 179.9 | 1.2 | -1.8 | -0.6 |
| $G_{-2}G_{-2}T_2$ | -58.1 | -56.5 | -178.7 | 1.9 | -1.3 | -1.0 |
| $G_{+1}G_{+1}T_1$ | 62.0 | 61.8 | -177.6 | 2.4 | 3.4 | 3.4 |
| $G_{+1}G_{+1}T_2$ | 61.7 | 61.2 | -175.3 | 3.6 | 2.1 | 1.9 |
| $G_{+2}G_{+2}T_1$ | 59.9 | 59.9 | -179.2 | 2.3 | 1.4 | 0.7 |
| $G_{+2}G_{+2}T_2$ | 57.9 | 58.7 | 173.2 | 1.7 | 1.2 | 0.5 |
| $G_{+1}G_{+1}G_{+1}$ | 61.8 | 62.0 | 62.0 | 4.1 | 4.3 | 4.0 |
| $G_{+2}G_{+2}G_{+2}$ | 60.3 | 60.4 | 60.3 | 0.8 | 0.7 | 0.8 |
| $G_{-1}G_{-1}G_{-1}$ | -67.1 | -67.5 | -67.4 | 1.8 | 1.6 | 1.9 |
| $G_{-2}G_{-2}G_{-2}$ | -57.6 | -57.5 | -57.4 | -1.9 | -1.6 | -1.7 |
| $G_{-1}G_{-1}G_{-2}$ | -66.2 | -68.1 | -56.8 | -1.0 | 3.0 | 0.4 |
| $G_{-1}G_{-1}G_{+2}$ | -66.8 | -66.2 | 56.4 | 2.7 | 2.4 | 0.0 |
| $G_{-1}G_{-2}G_{-2}$ | -67.2 | -56.5 | -57.3 | 0.4 | 0.6 | -1.5 |
| $G_{-1}G_{+2}G_{-2}$ | -65.2 | 57.5 | -55.7 | 2.2 | 1.2 | -2.7 |
| $G_{-1}G_{+2}G_{+2}$ | -66.1 | 56.6 | 59.9 | 2.1 | 0.4 | 0.9 |
| $G_{+2}G_{-2}G_{-2}$ | 57.2 | -54.1 | -57.4 | 1.2 | -1.9 | -2.6 |
| $G_{+2}G_{+2}G_{-2}$ | 57.8 | 59.7 | -53.8 | 1.0 | -1.9 | 0.5 |
| $G_{+1}G_{+2}G_{+2}$ | 61.8 | 57.4 | 59.8 | 2.1 | 0.8 | 0.9 |
| $G_{+1}G_{+1}G_{+2}$ | 62.4 | 61.9 | 57.2 | 0.4 | 1.6 | 3.1 |
| $G_{-1}G_{-1}G_{-1}b$ | -67.3 | -67.0 | -67.4 | -103.9 | -104.5 | -104.9 |
| $G_{-2}G_{-2}G_{-2}b$ | -58.4 | -58.3 | -58.4 | -107.9 | -108.6 | -107.6 |
| $G_{+2}G_{+2}G_{+2}b$ | 60.0 | 60.0 | 60.1 | -113.4 | -113.1 | -113.3 |
| $G_{-1}G_{+2}G_{-2}b$ | -65.5 | 57.6 | -56.7 | -106.6 | 2.6 | 2.6 |
| $G_{+1}G_{+2}G_{+2}b$ | 61.3 | 59.8 | 57.0 | 2.5 | 0.4 | -111.7 |

Table 3: The calculated chemical shifts of the linker and methoxy carbons of non-complexed Cryptophane-C in ppm. The column 'diff' is the difference between the average of 2' and the average of 1'.

| Conformer/Carbon | 1' | 1' | 1' | 2' | 2' | 2' | M | M | M | diff(2'-1') |
|---|---|---|---|---|---|---|---|---|---|---|
| $T_1T_1T_1$ | 75.1 | 75.1 | 74.8 | 75.3 | 75.2 | 75.0 | 56.3 | 56.1 | 56.0 | 0.2 |
| $T_2T_2T_2$ | 77.9 | 73.3 | 68.2 | 77.7 | 74.7 | 69.1 | 56.6 | 56.3 | 56.0 | 0.7 |
| $G_{+1}T_1T_1$ | 68.2 | 76.3 | 74.0 | 72.2 | 74.9 | 73.9 | 55.9 | 56.5 | 55.6 | 0.8 |
| $G_{+2}T_1T_1$ | 67.3 | 75.9 | 75.5 | 69.4 | 75.1 | 74.9 | 56.3 | 56.0 | 56.0 | 0.2 |
| $G_{+1}T_2T_2$ | 68.2 | 76.8 | 77.6 | 72.5 | 77.4 | 77.6 | 56.1 | 56.3 | 55.9 | 1.6 |
| $G_{+2}T_2T_2$ | 67.4 | 72.7 | 70.5 | 67.5 | 76.0 | 75.3 | 56.3 | 56.3 | 56.5 | 2.7 |
| $G_{-1}T_1T_1$ | 70.6 | 76.3 | 70.1 | 75.3 | 74.0 | 72.3 | 56.2 | 56.1 | 55.9 | 1.5 |
| $G_{-2}T_1T_1$ | 67.3 | 76.3 | 74.2 | 67.3 | 75.2 | 74.7 | 56.5 | 56.2 | 56.0 | -0.2 |
| $G_{-1}T_2T_2$ | 69.9 | 77.9 | 70.0 | 74.0 | 77.6 | 74.2 | 56.4 | 56.4 | 55.9 | 2.7 |
| $G_{-2}T_2T_2$ | 66.2 | 77.4 | 71.8 | 67.2 | 77.6 | 69.8 | 56.5 | 56.4 | 56.2 | -0.3 |
| $G_{-1}G_{-1}T_1$ | 70.8 | 70.2 | 76.5 | 75.5 | 75.2 | 74.3 | 56.5 | 56.0 | 55.9 | 2.5 |
| $G_{-1}G_{-1}T_2$ | 69.8 | 69.5 | 70.7 | 74.7 | 74.2 | 76.7 | 56.2 | 56.4 | 56.1 | 5.2 |
| $G_{-2}G_{-2}T_1$ | 69.2 | 67.9 | 75.9 | 67.6 | 67.0 | 75.0 | 56.6 | 56.2 | 56.2 | -1.1 |
| $G_{-2}G_{-2}T_2$ | 69.6 | 67.9 | 76.8 | 67.7 | 67.4 | 77.3 | 56.6 | 56.4 | 56.2 | -0.6 |
| $G_{+1}G_{+1}T_1$ | 69.7 | 69.2 | 75.6 | 72.7 | 72.7 | 74.1 | 56.4 | 55.8 | 55.4 | 1.7 |
| $G_{+1}G_{+1}T_2$ | 70.1 | 69.0 | 77.5 | 72.6 | 72.5 | 77.3 | 56.5 | 55.7 | 55.4 | 1.9 |
| $G_{+2}G_{+2}T_1$ | 67.5 | 67.6 | 75.7 | 70.4 | 68.8 | 74.6 | 56.3 | 56.3 | 56.3 | 1.0 |
| $G_{+2}G_{+2}T_2$ | 67.9 | 67.5 | 69.4 | 69.8 | 67.5 | 74.6 | 56.5 | 56.5 | 56.1 | 2.4 |
| $G_{+1}G_{+1}G_{+1}$ | 70.6 | 70.1 | 69.5 | 72.8 | 72.4 | 71.9 | 55.8 | 55.8 | 55.6 | 2.3 |
| $G_{+2}G_{+2}G_{+2}$ | 67.9 | 67.4 | 67.2 | 69.8 | 69.7 | 69.4 | 56.4 | 56.2 | 56.1 | 2.1 |
| $G_{-1}G_{-1}G_{-1}$ | 70.6 | 70.5 | 70.2 | 75.0 | 74.7 | 74.7 | 56.4 | 56.0 | 55.8 | 4.4 |
| $G_{-2}G_{-2}G_{-2}$ | 69.6 | 69.3 | 69.0 | 67.7 | 67.6 | 67.5 | 56.5 | 56.5 | 56.3 | -1.7 |
| $G_{-1}G_{-1}G_{-2}$ | 70.3 | 69.9 | 68.1 | 75.2 | 75.2 | 67.6 | 56.7 | 56.2 | 56.1 | 3.2 |
| $G_{-1}G_{-1}G_{+2}$ | 70.1 | 69.8 | 67.2 | 75.2 | 74.5 | 69.3 | 56.6 | 56.0 | 56.0 | 4.0 |
| $G_{-1}G_{-2}G_{-2}$ | 70.6 | 69.0 | 68.5 | 74.5 | 67.6 | 67.6 | 56.8 | 56.6 | 56.3 | 0.5 |
| $G_{-1}G_{+2}G_{-2}$ | 69.4 | 69.5 | 67.5 | 74.7 | 67.3 | 69.0 | 56.6 | 56.2 | 55.8 | 1.5 |
| $G_{-1}G_{+2}G_{+2}$ | 70.2 | 68.1 | 67.4 | 75.5 | 70.9 | 68.2 | 56.4 | 56.1 | 55.8 | 3.0 |
| $G_{+2}G_{-2}G_{-2}$ | 69.3 | 67.5 | 67.4 | 67.7 | 68.1 | 67.3 | 56.5 | 56.4 | 56.4 | -0.4 |
| $G_{+2}G_{+2}G_{-2}$ | 67.8 | 67.4 | 67.3 | 69.5 | 67.8 | 67.2 | 56.5 | 56.5 | 56.2 | 0.7 |
| $G_{+1}G_{+2}G_{+2}$ | 67.8 | 67.6 | 67.2 | 72.0 | 68.9 | 67.6 | 56.5 | 56.5 | 55.8 | 2.0 |
| $G_{+1}G_{+1}G_{+2}$ | 70.0 | 68.0 | 67.7 | 72.7 | 72.1 | 67.0 | 56.4 | 55.8 | 55.5 | 2.0 |
| $G_{-1}G_{-1}G_{-1}b$ | 70.7 | 70.6 | 70.4 | 74.9 | 74.7 | 74.7 | 62.4 | 62.4 | 62.2 | 4.2 |
| $G_{-2}G_{-2}G_{-2}b$ | 67.9 | 67.8 | 67.8 | 69.6 | 69.6 | 69.3 | 62.5 | 62.2 | 62.2 | 1.7 |
| $G_{+2}G_{+2}G_{+2}b$ | 68.0 | 67.3 | 67.2 | 69.6 | 69.5 | 69.2 | 62.3 | 62.3 | 62.1 | 1.9 |
| $G_{-1}G_{+2}G_{-2}b$ | 69.6 | 70.3 | 67.4 | 75.2 | 69.0 | 67.3 | 62.3 | 56.2 | 56.1 | 1.4 |
| $G_{+1}G_{+2}G_{+2}b$ | 68.4 | 67.9 | 67.4 | 72.2 | 69.0 | 67.8 | 62.4 | 56.3 | 55.8 | 1.8 |

Table 4: The relative energies of chloroform complexed Cryptophane-C in Kj/mol. Lower case 'e' denotes the energies obtained by the basis set 6-31G(d), and upper case E denotes the energies obtained by single point calculations at the basis set 6-311+G(2d,p). 'EE' and 'ee' contain the zero point corrections and the thermal corrections. 'EEE' and 'eee' contain the van der Waals(vdW) correction too. The column 'vdW' is the absolute value of the van der Waals correction in Kj/mol. The abbreviation 'deg' is the degeneracy of the conformer used for the Boltzmann distribution. The conformers labeled 'b' were optimized with different methoxy group dihedral (3"-2"-O-M) see Table 5.

| Conformer/Energy | deg | e | E | ee | EE | eee | EEE | vdW |
|---|---|---|---|---|---|---|---|---|
| $T_1T_1T_1(1)$ | 1 | 13.6 | 21.7 | 4.9 | 13.0 | 24.1 | 32.2 | -595.3 |
| $T_1T_1T_1(2)$ | 1 | 16.6 | 24.0 | 3.5 | 10.9 | 27.4 | 34.7 | -590.7 |
| $T_1T_1T_2(1)$ | 3 | 15.9 | 24.9 | 5.6 | 14.6 | 24.8 | 33.9 | -595.3 |
| $T_1T_2T_2(1)$ | 3 | 18.6 | 27.8 | 5.9 | 15.1 | 23.9 | 33.1 | -596.6 |
| $T_2T_2T_2(1)$ | 1 | 21.7 | 32.2 | 6.7 | 17.2 | 26.4 | 36.9 | -594.8 |
| $T_2T_2T_2(2)$ | 1 | 23.6 | 34.7 | 10.1 | 21.2 | 30.1 | 41.2 | -594.5 |
| $G_{+1}T_1T_1(1)$ | 3 | 14.4 | 23.4 | 10.5 | 19.6 | 19.0 | 28.1 | -606.0 |
| $G_{+1}T_1T_1(2)$ | 3 | 19.3 | 27.3 | 14.7 | 22.8 | 25.6 | 33.6 | -603.7 |
| $G_{-1}T_1T_1(1)$ | 3 | 16.5 | 23.3 | 13.8 | 20.6 | 17.9 | 24.7 | -610.5 |
| $G_{-1}T_1T_1(2)$ | 3 | 18.2 | 24.0 | 18.8 | 24.5 | 19.2 | 25.0 | -614.1 |
| $G_{+2}T_1T_1(1)$ | 3 | 8.7 | 14.1 | 4.8 | 10.1 | 17.4 | 22.7 | -601.9 |
| $G_{+2}T_1T_1(2)$ | 3 | 8.9 | 13.8 | 1.3 | 6.3 | 15.5 | 20.4 | -600.4 |
| $G_{-2}T_1T_1(1)$ | 3 | 16.1 | 21.7 | 14.6 | 20.2 | 20.4 | 26.0 | -608.7 |
| $G_{-2}T_1T_1(2)$ | 3 | 16.4 | 21.5 | 14.4 | 19.4 | 19.3 | 24.3 | -609.6 |
| $G_{+1}T_2T_2(1)$ | 3 | 20.2 | 30.9 | 14.3 | 25.0 | 23.8 | 34.5 | -605.0 |
| $G_{+1}T_2T_2(2)$ | 3 | 21.8 | 32.6 | 15.7 | 26.5 | 26.1 | 36.9 | -604.1 |
| $G_{-1}T_2T_2(1)$ | 3 | 20.9 | 29.7 | 13.7 | 22.4 | 17.3 | 26.0 | -610.9 |
| $G_{-1}T_2T_2(2)$ | 3 | 25.4 | 34.0 | 17.3 | 25.9 | 20.5 | 29.1 | -611.3 |
| $G_{+2}T_2T_2(1)$ | 3 | 12.4 | 19.7 | 5.0 | 12.3 | 16.4 | 23.7 | -603.2 |
| $G_{+2}T_2T_2(2)$ | 3 | 14.0 | 21.7 | 4.7 | 12.4 | 17.5 | 25.3 | -601.7 |
| $G_{-2}T_2T_2(1)$ | 3 | 21.1 | 27.7 | 15.4 | 21.9 | 20.8 | 27.4 | -609.1 |
| $G_{-2}T_2T_2(2)$ | 3 | 22.4 | 29.5 | 13.7 | 20.8 | 19.0 | 26.1 | -609.3 |
| $G_{+1}G_{+1}T_1(1)$ | 3 | 17.9 | 27.6 | 15.6 | 25.3 | 18.3 | 28.0 | -611.8 |
| $G_{+1}G_{+1}T_1(2)$ | 3 | 23.1 | 32.1 | 19.5 | 28.6 | 25.0 | 34.0 | -609.1 |
| $G_{-1}G_{-1}T_1(1)$ | 3 | 23.0 | 28.5 | 23.1 | 28.6 | 15.1 | 20.6 | -622.6 |
| $G_{-1}G_{-1}T_1(2)$ | 3 | 30.3 | 34.8 | 34.5 | 39.0 | 29.2 | 33.6 | -619.9 |
| $G_{+1}G_{+1}T_2(1)$ | 3 | 21.3 | 31.7 | 20.3 | 30.6 | 22.9 | 33.2 | -612.0 |
| $G_{+1}G_{+1}T_2(2)$ | 3 | 23.2 | 33.4 | 22.5 | 32.6 | 23.7 | 33.8 | -613.4 |
| $G_{-1}G_{-1}T_2(1)$ | 3 | 24.4 | 30.7 | 23.2 | 29.5 | 14.0 | 20.3 | -623.7 |
| $G_{-1}G_{-1}T_2(2)$ | 3 | 31.9 | 37.6 | 32.0 | 37.7 | 24.4 | 30.1 | -622.1 |
| $G_{+2}G_{+2}T_1(1)$ | 3 | 5.1 | 7.4 | 1.5 | 3.8 | 9.1 | 11.4 | -607.0 |
| $G_{+2}G_{+2}T_1(2)$ | 3 | 3.9 | 6.3 | 0.7 | 3.2 | 7.9 | 10.3 | -607.4 |

Table 4 continued

| Conformer/Energy | deg | e | E | ee | EE | eee | EEE | vdW |
|---|---|---|---|---|---|---|---|---|
| $G_{-2}G_{-2}T_1(1)$ | 3 | 23.7 | 26.0 | 24.5 | 26.8 | 19.1 | 21.5 | -619.8 |
| $G_{-2}G_{-2}T_1(2)$ | 3 | 20.8 | 23.5 | 23.0 | 25.7 | 17.8 | 20.6 | -619.7 |
| $G_{+2}G_{+2}T_2(1)$ | 3 | 5.8 | 9.1 | 4.4 | 7.7 | 10.2 | 13.4 | -608.8 |
| $G_{+2}G_{+2}T_2(2)$ | 3 | 6.1 | 10.2 | 2.8 | 6.9 | 7.9 | 12.0 | -609.5 |
| $G_{-2}G_{-2}T_2(1)$ | 3 | 25.3 | 28.0 | 24.3 | 27.0 | 18.8 | 21.5 | -620.0 |
| $G_{-2}G_{-2}T_2(2)$ | 3 | 23.6 | 27.5 | 22.8 | 26.6 | 14.8 | 18.6 | -622.6 |
| $G_{+1}G_{+1}G_{+1}(1)$ | 1 | 24.4 | 34.8 | 21.8 | 32.2 | 18.2 | 28.6 | -618.1 |
| $G_{+1}G_{+1}G_{+1}(2)$ | 1 | 28.5 | 38.0 | 28.1 | 37.7 | 25.4 | 35.0 | -617.2 |
| $G_{-1}G_{-1}G_{-1}(1)$ | 1 | 31.0 | 34.9 | 35.8 | 39.8 | 15.2 | 19.1 | -635.2 |
| $G_{-1}G_{-1}G_{-1}(2)$ | 1 | 42.9 | 44.8 | 46.9 | 48.8 | 32.4 | 34.3 | -629.1 |
| $G_{+2}G_{+2}G_{+2}(1)$ | 1 | 2.0 | 0.9 | 1.8 | 0.7 | 3.6 | 2.5 | -612.8 |
| $G_{+2}G_{+2}G_{+2}(2)$ | 1 | 0.0 | 0.0 | 0.0 | 0.0 | 0.0 | 0.0 | -614.6 |
| $G_{-2}G_{-2}G_{-2}(1)$ | 1 | 33.1 | 30.8 | 37.2 | 34.9 | 24.0 | 21.7 | -627.8 |
| $G_{-2}G_{-2}G_{-2}(2)$ | 1 | 27.3 | 26.7 | 30.8 | 30.2 | 12.8 | 12.2 | -632.6 |
| $G_{+1}T_1T_2(1)$ | 3 | 16.8 | 25.9 | 12.1 | 21.2 | 22.0 | 31.1 | -604.7 |
| $G_{+1}T_1T_2(2)$ | 3 | 17.8 | 27.1 | 12.4 | 21.7 | 16.6 | 25.9 | -610.3 |
| $G_{+1}G_{-2}T_1(1)$ | 3 | 21.5 | 27.1 | 20.9 | 26.4 | 17.8 | 23.3 | -617.7 |
| $G_{+1}G_{-2}T_1(2)$ | 3 | 25.0 | 30.5 | 21.5 | 26.9 | 22.0 | 27.5 | -614.0 |
| $G_{+1}G_{-2}T_2(1)$ | 3 | 24.2 | 30.4 | 23.8 | 30.0 | 20.5 | 26.7 | -617.9 |
| $G_{+1}G_{-2}T_2(2)$ | 3 | 25.7 | 32.4 | 24.6 | 31.2 | 21.8 | 28.5 | -617.3 |
| $G_{+2}G_{-2}T_1(1)$ | 3 | 15.2 | 17.5 | 11.9 | 14.2 | 13.2 | 15.4 | -613.3 |
| $G_{+2}G_{-2}T_1(2)$ | 3 | 13.2 | 16.2 | 13.0 | 16.1 | 12.6 | 15.6 | -615.0 |
| $G_{+1}G_{+2}G_{+2}(1)$ | 3 | 8.9 | 11.4 | 12.0 | 14.5 | 10.1 | 12.5 | -616.5 |
| $G_{+1}G_{+2}G_{+2}(2)$ | 3 | 8.1 | 11.3 | 9.9 | 13.1 | 5.6 | 8.9 | -618.8 |
| $G_{+2}G_{+2}G_{-2}(1)$ | 3 | 12.2 | 10.9 | 15.1 | 13.8 | 7.6 | 6.3 | -622.1 |
| $G_{+2}G_{+2}G_{-2}(2)$ | 3 | 8.7 | 8.9 | 14.6 | 14.8 | 5.2 | 5.4 | -624.0 |
| $G_{+2}G_{-2}G_{-2}(1)$ | 3 | 22.0 | 20.3 | 27.2 | 25.5 | 14.0 | 12.3 | -627.8 |
| $G_{+2}G_{-2}G_{-2}(2)$ | 3 | 18.1 | 18.5 | 24.1 | 24.5 | 9.2 | 9.6 | -629.5 |
| $G_{+2}G_{+2}G_{+2}(1)b$ | 1 | 20.6 | 21.0 | 14.3 | 14.6 | 19.4 | 19.8 | -609.5 |
| $G_{+2}G_{+2}G_{+2}(2)b$ | 1 | 17.6 | 19.6 | 11.9 | 13.9 | 14.8 | 16.8 | -611.7 |

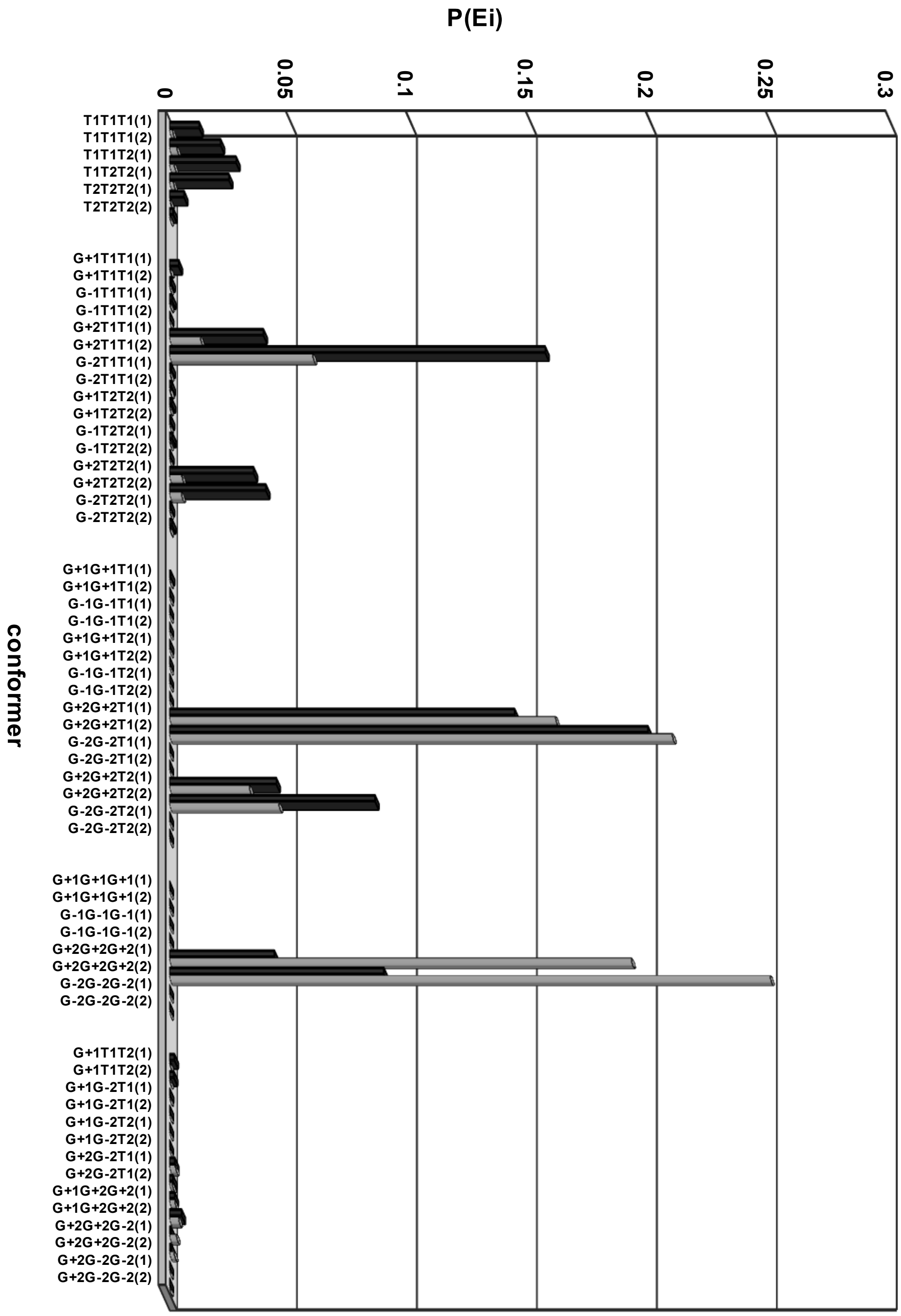


Figure S2: The Boltzmann distribution of conformers of chloroform complexed cryptophane-C based on the energies in column ee(box) and EE(cylinder) in Table 4.

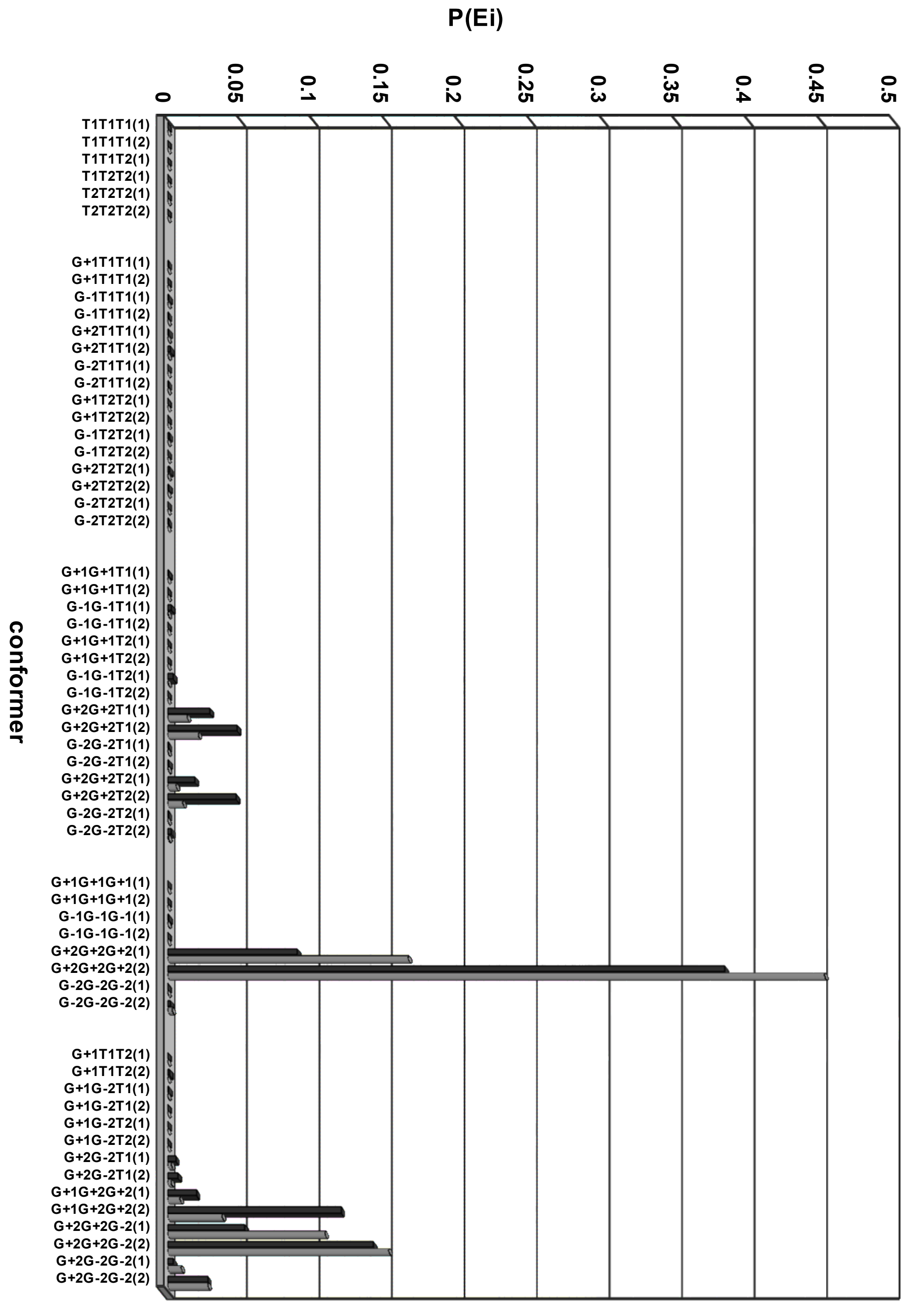


Figure S3: The Boltzmann distribution of conformers of chloroform complexed cryptophane-C based on the energies in column eee(box) and EEE(cylinder) in Table 4.

Table 5: The dihedral angle of the linkers and methoxy groups of chloroform complexed Cryptophane-C. The conformers labeled by 'b' have different dihedral angle of the methoxy groups.

| Conformer/Dihedral | O-1'-2'-O | O-1'-2'-O | O-1'-2'-O | 3"-2"-O-M | 3"-2"-O-M | 3"-2"-O-M |
|---|---|---|---|---|---|---|
| $T_1T_1T_1(1)$ | 178.7 | 179.2 | 179.3 | 1.4 | 2.0 | 2.1 |
| $T_1T_1T_1(2)$ | -174.8 | -172.6 | -172.4 | 1.9 | 0.9 | 0.5 |
| $T_1T_1T_2(1)$ | -168.8 | -178.9 | 179.9 | -0.2 | 0.8 | 2.8 |
| $T_1T_2T_2(1)$ | -169.9 | -170.9 | -178.5 | -0.1 | 0.1 | 3.2 |
| $T_2T_2T_2(1)$ | -168.9 | -169.4 | -169.0 | -0.6 | 0.2 | -0.4 |
| $T_2T_2T_2(2)$ | -168.2 | -167.9 | -167.9 | -0.7 | -0.2 | -0.6 |
| $G_{+1}T_1T_1(1)$ | 66.3 | -177.4 | 179.0 | 3.0 | 2.0 | 3.6 |
| $G_{+1}T_1T_1(2)$ | 65.9 | -177.5 | 177.7 | 1.6 | 2.5 | 1.9 |
| $G_{-1}T_1T_1(1)$ | -71.0 | 179.9 | -179.0 | 1.9 | 0.6 | 1.9 |
| $G_{-1}T_1T_1(2)$ | -73.8 | -178.7 | -167.0 | 2.6 | 3.8 | 0.8 |
| $G_{+2}T_1T_1(1)$ | 61.9 | -178.4 | -179.2 | -0.4 | 1.2 | 2.4 |
| $G_{+2}T_1T_1(2)$ | 64.6 | -173.4 | -169.3 | -0.9 | 1.4 | 0.6 |
| $G_{-2}T_1T_1(1)$ | -57.1 | -177.7 | 179.8 | -0.6 | 1.0 | -0.6 |
| $G_{-2}T_1T_1(2)$ | -58.4 | -177.9 | -169.7 | 1.0 | 1.4 | 0.7 |
| $G_{+1}T_2T_2(1)$ | 66.0 | -169.7 | -169.7 | 1.1 | 0.7 | 0.2 |
| $G_{+1}T_2T_2(2)$ | 65.8 | -169.3 | -168.2 | 2.2 | -1.6 | 0.3 |
| $G_{-1}T_2T_2(1)$ | -68.9 | -170.1 | -172.0 | 2.6 | -1.3 | -0.6 |
| $G_{-1}T_2T_2(2)$ | -68.5 | -170.1 | -170.4 | -0.6 | 2.2 | -2.6 |
| $G_{+2}T_2T_2(1)$ | 63.2 | -167.7 | -169.5 | 0.6 | -0.2 | -0.5 |
| $G_{+2}T_2T_2(2)$ | 64.9 | -169.2 | -167.5 | -0.3 | -0.5 | -1.8 |
| $G_{-2}T_2T_2(1)$ | -57.1 | -171.0 | -171.4 | -0.6 | 0.9 | -1.2 |
| $G_{-2}T_2T_2(2)$ | -57.0 | -171.5 | -170.4 | -2.7 | 0.1 | -0.4 |
| $G_{+1}G_{+1}T_1(1)$ | 67.2 | 68.1 | -176.9 | 1.8 | 2.0 | 2.1 |
| $G_{+1}G_{+1}T_1(2)$ | 67.5 | 66.3 | -175.8 | 2.7 | 1.9 | 1.8 |
| $G_{-1}G_{-1}T_1(1)$ | -71.8 | -70.5 | -179.8 | 1.7 | 2.1 | 0.9 |
| $G_{-1}G_{-1}T_1(2)$ | -71.2 | -70.2 | -175.2 | 1.4 | 3.3 | -0.2 |
| $G_{+1}G_{+1}T_2(1)$ | 68.6 | 67.1 | -169.6 | 2.0 | 1.7 | -0.4 |
| $G_{+1}G_{+1}T_2(2)$ | 65.6 | 67.5 | -169.4 | 1.4 | 3.2 | -1.1 |
| $G_{-1}G_{-1}T_2(1)$ | -71.1 | -69.9 | -174.1 | 1.8 | 2.4 | -0.8 |
| $G_{-1}G_{-1}T_2(2)$ | -70.1 | -69.8 | -173.4 | 2.7 | 2.7 | -3.1 |
| $G_{+2}G_{+2}T_1(1)$ | 64.5 | 63.4 | -177.0 | 0.7 | -0.2 | -0.5 |
| $G_{+2}G_{+2}T_1(2)$ | 65.5 | 65.2 | -170.2 | -2.0 | -1.8 | 0.1 |

Table 5 continued

| Conformer/Dihedral | O-1'-2'-O | O-1'-2'-O | O-1'-2'-O | 3"-2"-O-M | 3"-2"-O-M | 3"-2"-O-M |
|---|---|---|---|---|---|---|
| $G_{-2}G_{-2}T_1(1)$ | -57.5 | -58.4 | -177.2 | -0.5 | 0.3 | 1.2 |
| $G_{-2}G_{-2}T_1(2)$ | -58.7 | -59.1 | -174.7 | -0.4 | 0.3 | 0.8 |
| $G_{+2}G_{+2}T_2(1)$ | 64.5 | 64.7 | -168.6 | -1.1 | -1.0 | -0.5 |
| $G_{+2}G_{+2}T_2(2)$ | 66.2 | 65.0 | -168.2 | -1.1 | -1.5 | -1.7 |
| $G_{-2}G_{-2}T_2(1)$ | -57.8 | -59.0 | -171.9 | 0.0 | -2.0 | -0.5 |
| $G_{-2}G_{-2}T_2(2)$ | -58.8 | -59.2 | -171.7 | -1.1 | 0.7 | -2.5 |
| $G_{+1}G_{+1}G_{+1}(1)$ | 68.4 | 68.8 | 68.4 | 2.0 | 1.7 | 2.3 |
| $G_{+1}G_{+1}G_{+1}(2)$ | 67.7 | 67.3 | 67.5 | 3.0 | 3.1 | 2.5 |
| $G_{-1}G_{-1}G_{-1}(1)$ | -71.9 | -71.6 | -71.7 | 2.6 | 1.7 | 2.1 |
| $G_{-1}G_{-1}G_{-1}(2)$ | -70.6 | -71.1 | -70.2 | 1.0 | 2.4 | 2.9 |
| $G_{+2}G_{+2}G_{+2}(1)$ | 65.9 | 66.1 | 65.6 | -1.0 | -1.4 | -1.5 |
| $G_{+2}G_{+2}G_{+2}(2)$ | 66.9 | 66.7 | 66.7 | -1.6 | -1.4 | -1.3 |
| $G_{-2}G_{-2}G_{-2}(1)$ | -59.7 | -60.3 | -60.1 | -1.1 | -1.4 | -1.1 |
| $G_{-2}G_{-2}G_{-2}(2)$ | -60.8 | -61.0 | -61.0 | -0.9 | -0.4 | -0.5 |
| $G_{+1}T_1T_2(1)$ | 66.3 | -177.1 | 171.1 | 3.0 | 1.7 | 0.1 |
| $G_{+1}T_1T_2(2)$ | 65.3 | -167.4 | -167.4 | 0.7 | 2.3 | -0.8 |
| $G_{+1}G_{-2}T_1(1)$ | 67.6 | -57.0 | -178.0 | -0.2 | 0.5 | 0.7 |
| $G_{+1}G_{-2}T_1(2)$ | 64.7 | -57.8 | -174.7 | 0.1 | 0.8 | 1.3 |
| $G_{+1}G_{-2}T_2(1)$ | 66.2 | -57.9 | -171.2 | 0.1 | 0.5 | -1.4 |
| $G_{+1}G_{-2}T_2(2)$ | 65.0 | -58.2 | -170.1 | 0.8 | 0.5 | -2.7 |
| $G_{+2}G_{-2}T_1(1)$ | 64.3 | -56.1 | -177.7 | -1.5 | 0.5 | 1.1 |
| $G_{+2}G_{-2}T_1(2)$ | 65.2 | -57.6 | -176.4 | 1.4 | -2.4 | 0.2 |
| $G_{+1}G_{+2}G_{+2}(1)$ | 70.1 | 64.4 | 64.4 | 1.3 | -0.6 | -1.0 |
| $G_{+1}G_{+2}G_{+2}(2)$ | 66.9 | 66.5 | 66.4 | -1.3 | 2.0 | -1.8 |
| $G_{+2}G_{+2}G_{-2}(1)$ | 63.8 | 66.4 | -58.2 | 0.1 | -1.2 | -2.4 |
| $G_{+2}G_{+2}G_{-2}(2)$ | 65.7 | 67.2 | -58.4 | -1.8 | -2.4 | -0.3 |
| $G_{+2}G_{-2}G_{-2}(1)$ | 64.2 | -60.7 | -58.2 | -0.8 | -0.8 | -1.9 |
| $G_{+2}G_{-2}G_{-2}(2)$ | 64.7 | -60.2 | -58.5 | -2.9 | -1.2 | 0.4 |
| $G_{+2}G_{+2}G_{+2}(1)b$ | 65.3 | 65.4 | 65.2 | -116.1 | -115.8 | -116.6 |
| $G_{+2}G_{+2}G_{+2}(2)b$ | 66.0 | 65.9 | 66.3 | -116.6 | -115.7 | -115.9 |

Table 6: The calculated chemical shifts of the linker and methoxy carbons of chloroform complexed Cryptophane-C in ppm. The column 'diff' is the difference between the average of 2' and the average of 1'.

| Conformer/Carbon | 1' | 1' | 1' | 2' | 2' | 2' | M | M | M | diff(2'-1') |
|---|---|---|---|---|---|---|---|---|---|---|
| $T_1T_1T_1(1)$ | 76.0 | 76.0 | 75.3 | 74.6 | 74.3 | 74.0 | 56.2 | 56.0 | 55.8 | -1.5 |
| $T_1T_1T_1(2)$ | 73.0 | 72.6 | 72.2 | 73.6 | 73.0 | 72.5 | 56.1 | 56.1 | 55.8 | 0.4 |
| $T_1T_1T_2(1)$ | 77.0 | 75.5 | 74.9 | 77.1 | 73.9 | 73.9 | 56.2 | 56.1 | 56.0 | -0.8 |
| $T_1T_2T_2(1)$ | 77.1 | 75.7 | 75.6 | 76.7 | 76.7 | 73.8 | 56.2 | 55.9 | 55.9 | -0.4 |
| $T_2T_2T_2(1)$ | 77.3 | 77.2 | 76.9 | 76.8 | 76.7 | 76.7 | 56.4 | 56.0 | 56.0 | -0.4 |
| $T_2T_2T_2(2)$ | 76.5 | 76.7 | 76.7 | 77.6 | 77.4 | 77.4 | 56.1 | 56.1 | 56.2 | 0.8 |
| $G_{+1}T_1T_1(1)$ | 70.2 | 75.7 | 75.6 | 73.0 | 73.9 | 73.8 | 55.6 | 55.8 | 55.9 | -0.3 |
| $G_{+1}T_1T_1(2)$ | 70.1 | 76.1 | 75.7 | 74.1 | 73.8 | 73.6 | 55.4 | 55.9 | 56.0 | -0.1 |
| $G_{-1}T_1T_1(1)$ | 69.9 | 76.0 | 75.9 | 74.0 | 73.5 | 75.1 | 56.0 | 55.8 | 55.7 | 0.3 |
| $G_{-1}T_1T_1(2)$ | 70.1 | 76.0 | 70.8 | 74.2 | 73.5 | 73.5 | 56.3 | 55.9 | 55.7 | 1.4 |
| $G_{+2}T_1T_1(1)$ | 67.9 | 76.7 | 76.3 | 69.7 | 74.6 | 74.1 | 56.3 | 55.9 | 55.7 | -0.8 |
| $G_{+2}T_1T_1(2)$ | 68.4 | 72.7 | 72.3 | 70.1 | 73.3 | 73.2 | 56.4 | 56.2 | 56.0 | 1.1 |
| $G_{-2}T_1T_1(1)$ | 69.2 | 76.4 | 75.3 | 67.6 | 74.9 | 73.6 | 56.3 | 55.9 | 55.9 | -1.6 |
| $G_{-2}T_1T_1(2)$ | 68.4 | 77.0 | 71.7 | 67.5 | 74.5 | 73.6 | 56.2 | 56.0 | 55.8 | -0.5 |
| $G_{+1}T_2T_2(1)$ | 70.7 | 77.4 | 76.7 | 73.5 | 76.4 | 77.0 | 56.1 | 56.0 | 55.6 | 0.7 |
| $G_{+1}T_2T_2(2)$ | 70.0 | 77.0 | 76.4 | 72.9 | 77.2 | 77.1 | 56.3 | 56.0 | 55.7 | 1.3 |
| $G_{-1}T_2T_2(1)$ | 73.8 | 77.4 | 76.5 | 70.1 | 77.1 | 76.6 | 56.1 | 55.8 | 55.7 | -1.3 |
| $G_{-1}T_2T_2(2)$ | 69.6 | 76.4 | 76.9 | 73.7 | 77.2 | 76.7 | 56.2 | 56.1 | 55.9 | 1.6 |
| $G_{+2}T_2T_2(1)$ | 67.9 | 76.9 | 76.9 | 68.9 | 77.1 | 76.9 | 56.5 | 56.1 | 55.9 | 0.4 |
| $G_{+2}T_2T_2(2)$ | 68.3 | 76.7 | 76.6 | 69.6 | 77.3 | 77.3 | 56.4 | 56.0 | 56.0 | 0.9 |
| $G_{-2}T_2T_2(1)$ | 68.9 | 77.7 | 76.1 | 67.6 | 76.9 | 76.4 | 56.2 | 56.1 | 55.8 | -0.6 |
| $G_{-2}T_2T_2(2)$ | 68.8 | 76.0 | 75.9 | 67.5 | 77.4 | 77.0 | 56.2 | 56.2 | 56.1 | 0.4 |
| $G_{+1}G_{+1}T_1(1)$ | 72.3 | 70.7 | 76.3 | 74.0 | 73.3 | 73.9 | 56.2 | 55.7 | 55.4 | 0.6 |
| $G_{+1}G_{+1}T_1(2)$ | 70.2 | 70.7 | 76.5 | 73.2 | 73.1 | 74.0 | 55.9 | 55.6 | 55.6 | 1.0 |
| $G_{-1}G_{-1}T_1(1)$ | 70.4 | 69.4 | 76.1 | 73.9 | 73.9 | 74.1 | 55.7 | 55.6 | 55.6 | 2.0 |
| $G_{-1}G_{-1}T_1(2)$ | 70.4 | 70.3 | 74.5 | 74.0 | 73.4 | 74.9 | 56.1 | 55.9 | 55.7 | 2.4 |
| $G_{+1}G_{+1}T_2(1)$ | 71.0 | 70.6 | 77.2 | 73.5 | 73.5 | 76.4 | 56.1 | 55.3 | 55.2 | 1.5 |
| $G_{+1}G_{+1}T_2(2)$ | 70.8 | 69.9 | 76.7 | 73.7 | 73.1 | 76.7 | 55.7 | 55.2 | 55.0 | 2.0 |
| $G_{-1}G_{-1}T_2(1)$ | 69.7 | 69.6 | 76.5 | 74.1 | 73.3 | 76.8 | 56.2 | 55.7 | 55.6 | 2.8 |
| $G_{-1}G_{-1}T_2(2)$ | 70.4 | 69.7 | 76.3 | 73.8 | 73.6 | 76.4 | 55.7 | 55.5 | 55.5 | 2.5 |
| $G_{+2}G_{+2}T_1(1)$ | 68.4 | 67.8 | 76.7 | 70.4 | 70.1 | 74.5 | 56.2 | 56.2 | 56.2 | 0.7 |
| $G_{+2}G_{+2}T_1(2)$ | 68.3 | 68.2 | 72.2 | 70.1 | 70.1 | 73.7 | 56.3 | 56.0 | 55.8 | 1.7 |

Table 6 continued

| Conformer/Carbon | 1’ | 1’ | 1’ | 2’ | 2’ | 2’ | M | M | M | diff(2’-1’) |
|---|---|---|---|---|---|---|---|---|---|---|
| $G_{-2}G_{-2}T_1(1)$ | 70.0 | 68.4 | 76.6 | 67.7 | 67.5 | 73.7 | 56.3 | 56.1 | 55.9 | -2.0 |
| $G_{-2}G_{-2}T_1(2)$ | 70.5 | 68.8 | 74.2 | 68.0 | 67.5 | 73.6 | 56.2 | 56.2 | 55.5 | -1.5 |
| $G_{+2}G_{+2}T_2(1)$ | 68.0 | 68.0 | 76.9 | 69.9 | 69.8 | 76.8 | 56.2 | 56.2 | 56.1 | 1.2 |
| $G_{+2}G_{+2}T_2(2)$ | 68.9 | 68.5 | 75.7 | 70.8 | 70.4 | 77.4 | 56.2 | 56.0 | 56.0 | 1.8 |
| $G_{-2}G_{-2}T_2(1)$ | 70.4 | 69.0 | 77.0 | 68.1 | 68.0 | 76.8 | 56.2 | 56.2 | 55.6 | -1.2 |
| $G_{-2}G_{-2}T_2(2)$ | 69.7 | 69.0 | 76.0 | 67.9 | 67.7 | 76.6 | 56.3 | 56.2 | 56.0 | -0.8 |
| $G_{+1}G_{+1}G_{+1}(1)$ | 71.5 | 71.4 | 71.3 | 74.2 | 73.5 | 73.5 | 55.5 | 55.1 | 55.1 | 2.3 |
| $G_{+1}G_{+1}G_{+1}(2)$ | 71.2 | 70.9 | 70.7 | 73.8 | 73.5 | 73.5 | 55.6 | 55.5 | 55.2 | 2.7 |
| $G_{-1}G_{-1}G_{-1}(1)$ | 70.3 | 69.9 | 69.7 | 74.3 | 74.1 | 73.8 | 55.8 | 55.8 | 55.7 | 4.1 |
| $G_{-1}G_{-1}G_{-1}(2)$ | 70.2 | 70.2 | 69.9 | 74.2 | 74.0 | 74.0 | 55.7 | 55.6 | 55.5 | 4.0 |
| $G_{+2}G_{+2}G_{+2}(1)$ | 68.4 | 68.3 | 68.0 | 71.2 | 71.2 | 70.8 | 56.2 | 56.1 | 56.0 | 2.8 |
| $G_{+2}G_{+2}G_{+2}(2)$ | 69.0 | 68.7 | 68.6 | 71.6 | 71.3 | 71.0 | 56.3 | 56.1 | 56.0 | 2.5 |
| $G_{-2}G_{-2}G_{-2}(1)$ | 70.5 | 69.7 | 69.7 | 68.2 | 68.0 | 68.0 | 56.2 | 56.1 | 56.1 | -1.9 |
| $G_{-2}G_{-2}G_{-2}(2)$ | 69.9 | 69.8 | 69.0 | 68.0 | 68.0 | 67.9 | 56.2 | 56.1 | 55.9 | -1.6 |
| $G_{+1}T_1T_2(1)$ | 70.5 | 76.5 | 75.9 | 73.3 | 76.9 | 73.9 | 55.2 | 56.1 | 55.9 | 0.4 |
| $G_{+1}T_1T_2(2)$ | 69.3 | 77.2 | 71.7 | 72.4 | 76.9 | 74.0 | 56.1 | 55.9 | 55.9 | 1.7 |
| $G_{+1}G_{-2}T_1(1)$ | 70.8 | 68.9 | 75.9 | 73.3 | 67.3 | 73.5 | 56.2 | 56.0 | 55.5 | -0.5 |
| $G_{+1}G_{-2}T_1(2)$ | 70.1 | 69.7 | 76.8 | 73.2 | 67.8 | 74.6 | 56.2 | 55.7 | 55.5 | -0.3 |
| $G_{+1}G_{-2}T_2(1)$ | 69.7 | 69.6 | 77.4 | 73.4 | 67.7 | 76.4 | 56.2 | 56.2 | 55.3 | 0.3 |
| $G_{+1}G_{-2}T_2(2)$ | 70.0 | 69.4 | 76.6 | 73.7 | 67.3 | 76.5 | 55.3 | 56.1 | 56.0 | 0.5 |
| $G_{+2}G_{-2}T_1(1)$ | 68.5 | 69.2 | 76.9 | 70.6 | 67.2 | 73.5 | 56.2 | 56.0 | 56.0 | -1.1 |
| $G_{+2}G_{-2}T_1(2)$ | 68.4 | 68.6 | 74.3 | 70.3 | 67.4 | 73.8 | 56.3 | 56.2 | 55.8 | 0.1 |
| $G_{+1}G_{+2}G_{+2}(1)$ | 69.9 | 69.7 | 68.0 | 73.4 | 70.3 | 68.2 | 56.2 | 56.1 | 55.4 | 1.4 |
| $G_{+1}G_{+2}G_{+2}(2)$ | 70.1 | 69.1 | 68.8 | 73.1 | 70.8 | 70.7 | 56.3 | 56.3 | 55.2 | 2.2 |
| $G_{+2}G_{+2}G_{-2}(1)$ | 69.1 | 68.8 | 68.1 | 71.3 | 70.3 | 67.8 | 56.2 | 56.2 | 56.1 | 1.1 |
| $G_{+2}G_{+2}G_{-2}(2)$ | 69.2 | 69.2 | 68.5 | 71.8 | 70.3 | 67.4 | 56.1 | 56.1 | 56.0 | 0.9 |
| $G_{+2}G_{-2}G_{-2}(1)$ | 70.1 | 68.2 | 68.1 | 69.9 | 67.9 | 67.6 | 56.3 | 56.3 | 55.9 | -0.3 |
| $G_{+2}G_{-2}G_{-2}(2)$ | 70.1 | 68.9 | 68.4 | 70.0 | 67.7 | 67.4 | 56.2 | 56.2 | 56.1 | -0.8 |
| $G_{+2}G_{+2}G_{+2}(1)b$ | 68.4 | 68.2 | 67.7 | 70.8 | 70.6 | 70.5 | 62.0 | 62.0 | 61.9 | 2.5 |
| $G_{+2}G_{+2}G_{+2}(2)b$ | 68.3 | 68.2 | 67.5 | 71.4 | 70.7 | 70.5 | 62.1 | 62.1 | 61.9 | 2.9 |

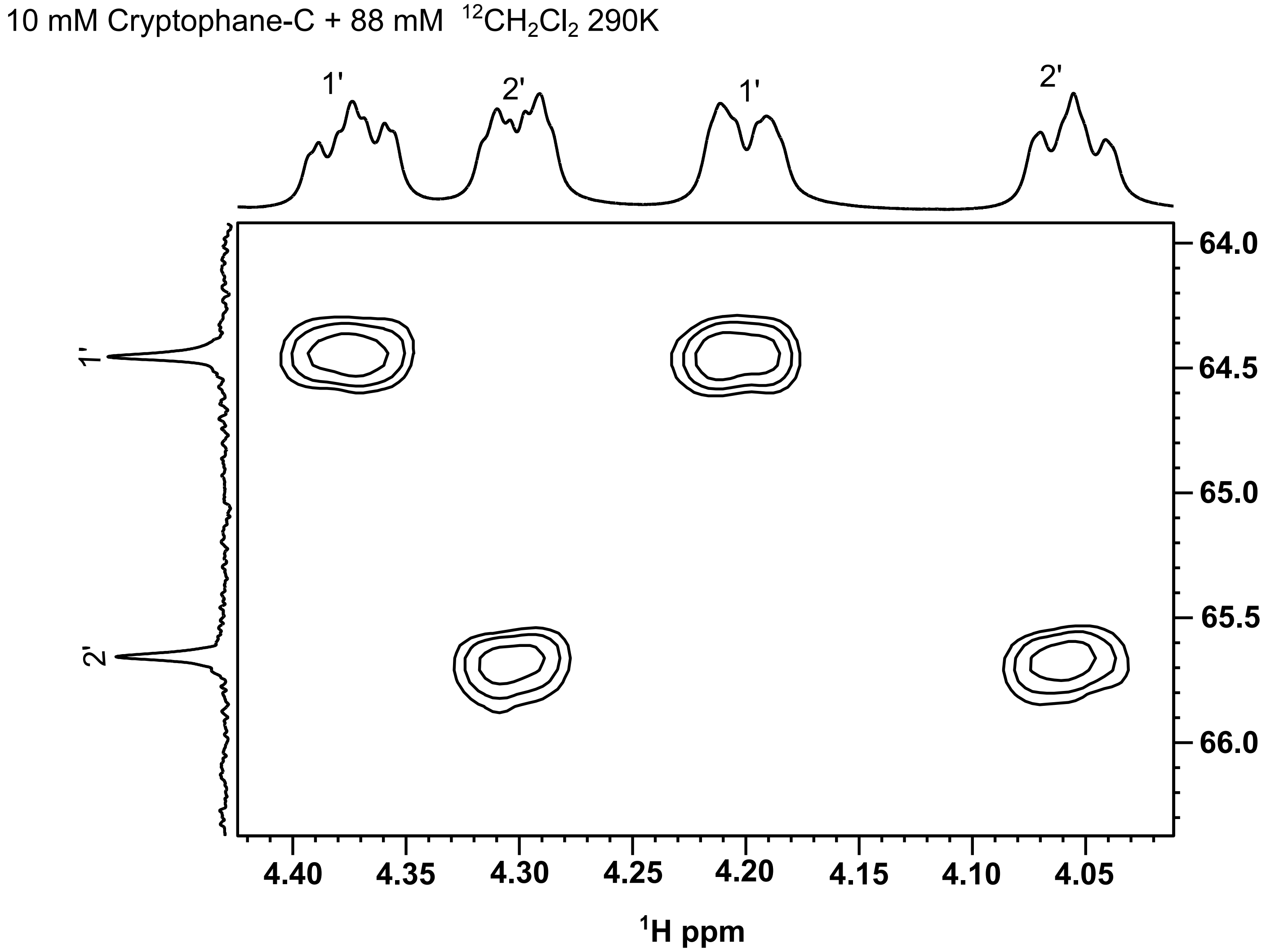


Figure S4: Part of the HSQC spectrum of the solution of 11 mM of Cryptophane-C and 88 mM $CH_2Cl_2$ at 290K and 600 MHz for $^1$H and 150 MHz for $^{13}$C
