# Supplementary figures and images for: Host–guest complexes between cryptophane-C and chloromethanes revisited
Source: Magn Reson Chem. 2012 Nov 7;51(1):19–31. doi: 10.1002/mrc.3898 (PMC3568900; doi:10.1002/mrc.3898)

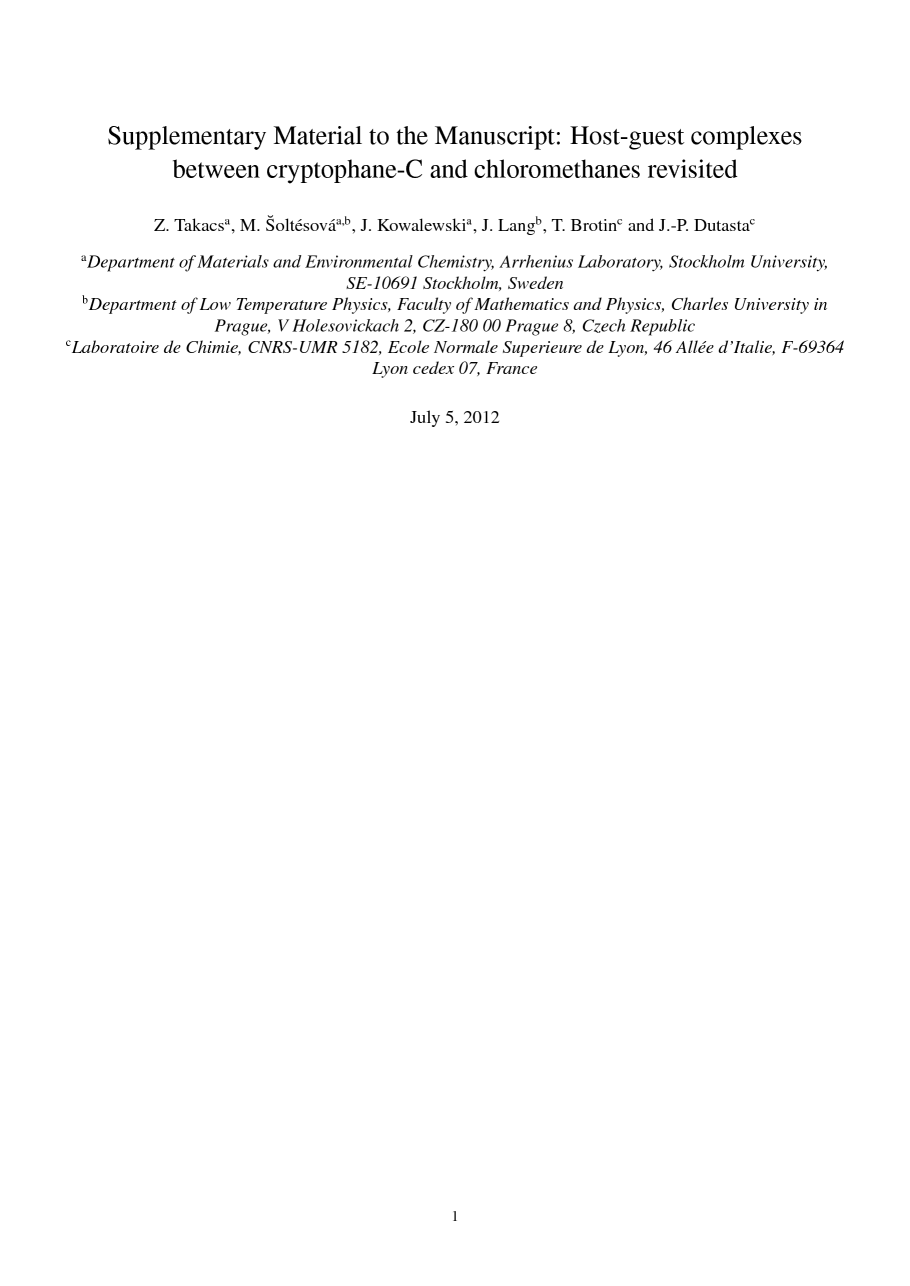

Supplement: Supplementary file 2 [file mrc0051-0019-SD2.png]
